# Plasmonic Enhanced Nonlinear Absorption of Tris(2-aminoethyl)amine (TREN) Functional Ag, Pt, and Pd Nanoparticle-GQDs Complexes and Their Evaluation as Potential Bioimaging Applications

**DOI:** 10.1021/acsabm.5c00750

**Published:** 2025-07-01

**Authors:** Bekir Asilcan Unlu, Esen Kirit, Dogantan Celik, Elif Akhuseyin Yildiz, Ahmet Karatay, Bahadir Boyacioglu, Hüseyin Ünver, Açelya Yilmazer, Mustafa Yıldız, Ayhan Elmali

**Affiliations:** † Department of Engineering Physics, Faculty of Engineering, 37504 Ankara University, 06100 Beşevler, Ankara, Türkiye; ‡ Graduate School of Natural and Applied Sciences, Ankara University, 06100 Ankara, Türkiye; § Department of Biomedical Engineering, Faculty of Engineering, Ankara University, 06830 Ankara, Türkiye; ∥ Stem Cell Institute, Ankara University, 06520 Ankara, Türkiye; ⊥ Institute of Artificial Intelligence, Ankara University, TR-06100 Ankara, Türkiye; # Vocational School of Health Services, Ankara University, Kecioren, 06290 Ankara, Türkiye; ∇ Department of Physics, Faculty of Science, Ankara University, 06100 Beşevler, Ankara, Türkiye; ○ Department of Chemistry, Faculty of Arts and Sciences, Çanakkale Onsekiz Mart University, TR-17100 Çanakkale, Türkiye

**Keywords:** tris(2-aminoethyl)amine (TREN), N-GQDs, metal nanocomposites, ultrafast spectroscopy, bioimaging, DFT

## Abstract

Tris (2-aminoethyl)­amine (TREN) functionalized N-doped graphene quantum dots (N-GQDs) and their Ag, Pd, and Pt nanocomposites were synthesized via a green one-step method and comprehensively characterized using FT-IR, UV–vis, TEM, and EDX. Spectroscopic analysis revealed π–π* transitions of C=C bonds (245–250 nm) and *n*–π* transitions of C=O and C=N bonds (334–350 nm), elucidating the materials’ optical properties. Photoluminescence studies revealed excitation wavelength-dependent emissions, indicating the presence of edge defect levels. Femtosecond transient absorption spectroscopy revealed a shortened excited-state lifetime upon incorporation of a metal atom into TREN-GQDs. Nonlinear absorption was explored by using the open-aperture Z-scan method, revealing enhanced performance upon incorporation of plasmonic nanoparticles. Ag-incorporated samples exhibited the highest NLA response due to plasmon-enhanced two-photon absorption. Notably, Pt-incorporated N-GQDs showed improved NLA and good biocompatibility with the intracellular fluorescence response, positioning them as promising candidates for bioimaging applications. Density functional theory (DFT) calculations, including gas-phase optimizations and aqueous simulations, confirmed alignment with experimental results, highlighting the enhanced stability and reactivity of nanocomposites. These findings highlight the potential of these materials in various applications, such as optical limiting, imaging, photovoltaics, and sensing applications.

## Graphical abstract

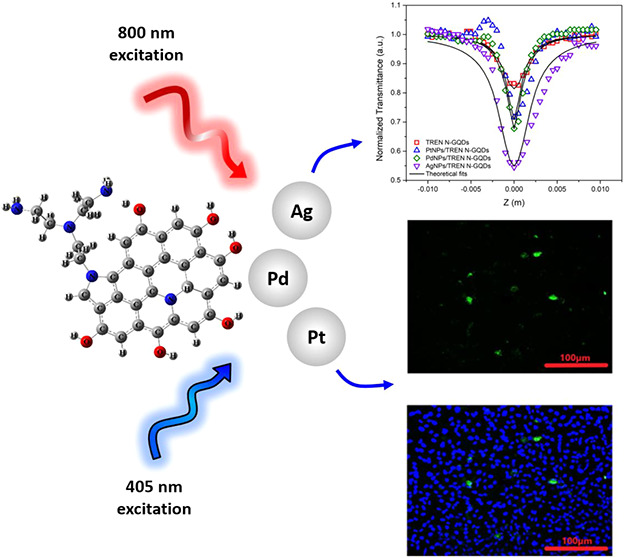

## 1 Introduction

Tris (2-aminoethyl) amine (TREN) is a colorless liquid, water-soluble, and highly basic compound consisting of a tertiary amine center and three dangling primary amine groups. It is used as a cross-linking agent in the synthesis of polyimine networks and as a tripodal ligand in coordination chemistry. TREN is a tetradentate ligand that forms stable complexes with transition metals in the (2^+^) and (3^+^) oxidation states. TREN is known to react very rapidly with aldehydes, especially in the presence of aromatic aldehydes, to form imines. Therefore, TREN is widely used in the preparation of polyimines.^1^ TREN-based tripodal ligand TRENOL was synthesized, and its coordination behavior with trivalent metal ions, Fe­(III) and Cr­(III), was investigated potentiometrically and spectrophotometrically, and it was suggested that it can be used as an iron detector.^2^ MgO, Al_2_O_3_, and Nb_2_O_5_ metal oxides were modified with tris (2-aminoethyl) amine by two methods to investigate their catalyst properties in Knoevenagel condensation between furfural and malonononitrile. TREN-modified MgO catalyst was found to show superior activity.^3^ A review is presented of tripodal Schiff base complexes of tris (2-aminoethyl)­amine, and several tripodal amines closely related to Cr­(II), Mn­(II), Mn­(III), Fe­(II), Fe­(III), and Co­(II) are presented.^3^

In recent years, the discovery of nanomaterials has generated significant interest in the field of optics with the search for new materials with unique optical properties and versatile functions. Among these emerging nanomaterials, graphene quantum dots (GQDs) have garnered considerable attention due to their extraordinary optical properties and intriguing quantum confinement effects.^4–6^ The optical properties of GQDs cover a wide spectral range, from ultraviolet to near-infrared wavelengths. These materials exhibit strong light absorption and photoluminescence properties with emission wavelengths that can be controlled through precise manipulation of their size, shape, surface, and edge chemistries.^7–9^ Such tunability offers unique opportunities to tailor GQD-based optical devices to meet specific application requirements in different fields.

GQDs have intriguing nonlinear optical properties, making them attractive for advanced applications in nonlinear optics and photonics. Their high nonlinear optical coefficients and ultrafast response times enable efficient generation and manipulation of nonlinear optical signals, paving the way for advances in areas such as frequency conversion,^10^ optical switching,^11–14^ and ultrafast photonics.^15–19^ Moreover, the inherent biocompatibility and low toxicity of GQDs make them promising candidates for bioimaging, biosensor, and biomedical applications, further expanding their scope from optical studies to medical studies.

More specifically, GQD nanostructures have applications in photodynamic therapy (PDT) used in the treatment of cancer cells. In order to penetrate deeper areas in patient tissues, the spectrum of agent nanostructures must be excited in the range of 750–1000 nm.^20^ It is possible to excite agent nanostructures in the near-infrared region of the spectrum by using a two-photon absorption mechanism (TPA). The two-photon absorption mechanism provides great advantages in PDT and biological imaging applications due to its deeper penetration, lower scattering, point application in treatment, and imaging capabilities.^21–23^ Thanks to the specified features of the two-photon absorption mechanism, treatment is provided with much less damage to healthy cells, and the location of tumor cells can be determined more clearly. By focusing the light on the target area and absorbing two photons, high-frequency emission enables the elimination of background signals. This distinction can be made if the emission frequency of unfocused light outside the target region is equal to or less than the excitation frequency. In order to be used in intracellular imaging applications, the absorption and emission properties of GQDs structures must be well understood and known.

GQDs can be easily doped with heteroatoms such as nitrogen, boron, and phosphorus to increase their quantum yields and fluorescence efficiency. In this context, N-doping has been found to be highly beneficial in modifying the intrinsic properties of GQDs.^24,25^ N-GQDs are widely used in fluorescence sensors to detect metal and nonmetal ions, small organic molecules, and biological macromolecules.^26,27^ Various methods have been developed for the synthesis of N-GQDs.^28–33^ Among these synthesis methods, hydrothermal synthesis is the most popular and simple, with the lowest cost.

Nitrogen-doped graphene quantum dots (N-GQDs) for biological applications and cancer therapy are highly studied due to their excellent biocompatibility and photoluminescence properties, as well as their ability to improve drug delivery and imaging in the context of cancer treatment and diagnosis.^34–37^ When lung adenocarcinoma cells (A549) were treated with AgNPs/PEI N-GQDs nanocomposites, material-specific fluorescence signals were obtained from the cytoplasm of the cells. Ag/PEI N-doped GQDs were shown to be promising nanosystems for bioimaging applications.^33^ Due to their different sizes and structures, nanoparticles such as Pt and Pd have been used to improve the sensitivity of therapeutic agents. To enhance the biocompatibility and cellular uptake of N-GQDs and Ag, Pd, and Pt nanocomposites, TREN N-GQDs/Ag, Pd, and Pt nanocomposite models were synthesized. These materials are intended to transform them into usable tools for both imaging and drug delivery.

In this study, TREN N-GQDs were synthesized from a one-step hydrothermal reaction of citric acid and tris­(2-aminoethyl)­amine (TREN) in water, and Ag-, Pd-, Pt-NPs/TREN N-GQDs nanocomposites were prepared from one-step hydrothermal reactions of synthesized TREN N-GQDs with AgNO_3_, Pd­(NO_3_)_2_, and K_2_PtCl_4_ in water (Scheme 1). TREN N-GQDs and Ag-, Pd-, and Pt-NPs/TREN N-GQDs nanocomposites were characterized by transmission electron microscopy (TEM), ultraviolet–visible spectroscopy (UV–vis), Fourier transform infrared spectroscopy (FT-IR), and energy-dispersive X-ray spectroscopy (EDX). The nonlinear optical features of the synthesized compounds were characterized utilizing femtosecond transient absorption spectroscopy and open-aperture Z-scan techniques. We also employed density functional theory (DFT) to comprehensively analyze the electronic structures of TREN N-GQDs and their nanocomposites with Ag, Pd, and Pt nanoparticles.

**Scheme 1:**
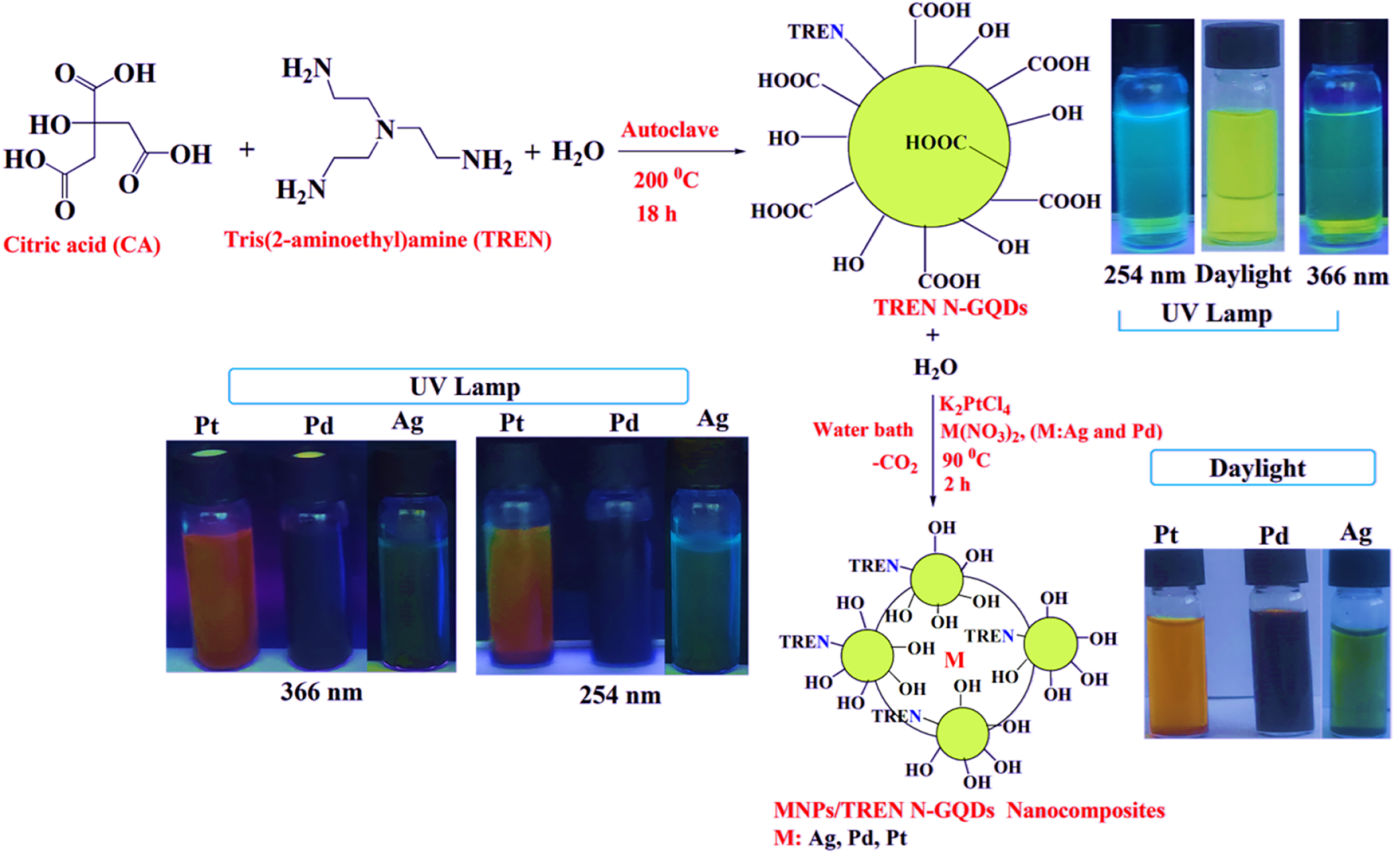
Synthesis of TREN N-GQDs and MNPs/TREN N-GQDs Nanocomposites

## 2 Materials and Methods

### 2.1 Materials

All chemicals, including citric acid (CA), silver nitrate (AgNO_3_), palladium­(II) nitrate dihydrate (Pd­(NO_3_)_2_2H_2_O), potassium tetrachloroplatinate­(II) (K_2_PtCl_4_), and tris­(2-aminoethyl)­amine (TREN) were sourced commercially from Sigma-Aldrich and used without further purification.

### 2.2 Synthesis of TREN N-GQDs and Ag-, Pd-, and Pt-NPs/TREN N-GQDs Nanocomposites

#### 2.2.1 Synthesis of TREN N-GQDs

Citric acid monohydrate (1.40 g, 6.66 mmol) and TREN (0.976 g, 6.68 mmol) were dissolved in 50 mL of deionized water and incubated in an autoclave at 200 °C for 18 h. After cooling to room temperature, the suspension products were centrifuged at 12,000 rpm for 10 min, and the supernatant was collected. TREN N-GQDs nanoparticles were washed twice with deionized water and once with acetone and dried in a vacuum oven at 50 °C. The obtained TREN N-GQDs nanoparticles were stored in a vacuum desiccator for further use.^28–33,38,39^

#### 2.2.2 Synthesis of Ag-, Pd-, and Pt-NPs/TREN N-GQDs Nanocomposites

For each metal nanocomposite synthesis, TREN N-GQDs (0.150 g) were added to a 250 mL round-bottomed flask, and 50 mL of water was added. Then, a 0.150 g solution of each metal salt (Ag, Pd, and Pt) in 50 mL of water was added to this solution, and the mixture was heated in a water bath at 90 °C for 2 h to form metal nanocomposites. After this time, the color of the solution changed from yellow to gray-black, and the metal cations (Ag^+^, Pd^2+^, and Pt^2+^) were completely reduced to their metal (M). The solution was filtered, and the solid was washed twice with deionized water and once with acetone. The powdered Ag-, Pd-, and Pt-NPs/TREN N-GQDs nanocomposites were dried in a vacuum oven and stored in a vacuum desiccator for further use.

### 2.3 Material Characterization

The UV–vis absorption spectra of the samples were recorded using a Shimadzu UV-1800 UV–vis absorption spectrophotometer. PL measurements were conducted by employing a PerkinElmer LS55 fluorescence spectrometer. TEM images and EDX patterns were obtained by a FEIZEISS EVO 40 (500 V to 30 kV) scanning electron microscope. The nonlinear absorption (NLA) properties were investigated by the open-aperture Z-scan method. Ultrafast laser at 800 nm wavelength with 1 ps pulse duration and 1 kHz frequency was used as a light source with a Gaussian intensity distribution. The laser beam was focused to a 20 μm waist size, and the samples were moved along the optical axis by using a motorized translation stage controlled by an Aerotech Unidex 511 motion controller. The transmitted beam was detected by a photodiode and processed with a boxcar integrator, and the transmittance was recorded and converted into normalized values.

### 2.4 DFT Method

In this section, DFT is used for a detailed analysis of several nanomaterials, including TREN N-GQDs and MNPs/TREN N-GQDs (M = Ag, Pd and Pt) nanocomposites. The main objective is to assess the sensitivity and selectivity of these materials by means of a study of their electronic properties. The analysis includes essential calculations such as optimizing geometry, analyzing electronic structure, charge distribution, and UV–vis analysis. These calculations offer a comprehensive understanding of the electronic, structural, thermal, optical, and energetic characteristics of these materials, thus establishing a foundation for their potential applications. The analyses were meticulously evaluated using Gaussview 5.0 software,^40^ which facilitated the visualization and interpretation of the complex data derived from the DFT calculations. This software enabled a profound comprehension of the electronic and structural properties of the nanomaterials under study.

To determine the most stable structural configurations of these theoretical models, optimization has been carried out using the DFT/B3LYP method with Gaussian 09W software.^41^ The use of the Lanl2dz basis set from Los Alamos National Laboratory ensured the accuracy of the calculations, which is particularly beneficial for transition metal-containing compounds such as quantum dots.^42,43^ This basis set also facilitated the determination of critical properties, such as the highest occupied molecular orbital (HOMO), lowest unoccupied molecular orbital (LUMO), and HOMO–LUMO energy gap. Additionally, we have computed the UV–vis range data using time-dependent density functional theory (TD-DFT),^44^ with all calculations performed using the same basis set.^45,46^ Our computational system faced performance and efficiency limitations due to the considerable size of the nanocomposites under investigation. This made it difficult to effectively perform the necessary calculations. To overcome these challenges, as in our previous studies,^33,47^ we constructed molecular models that retained structural similarities to the original composites, using Gaussview 5.0 software. This approach allowed us to manage the computational requirements while ensuring the integrity and relevance of the electronic property analyses (Scheme 2).

**Scheme 2:**
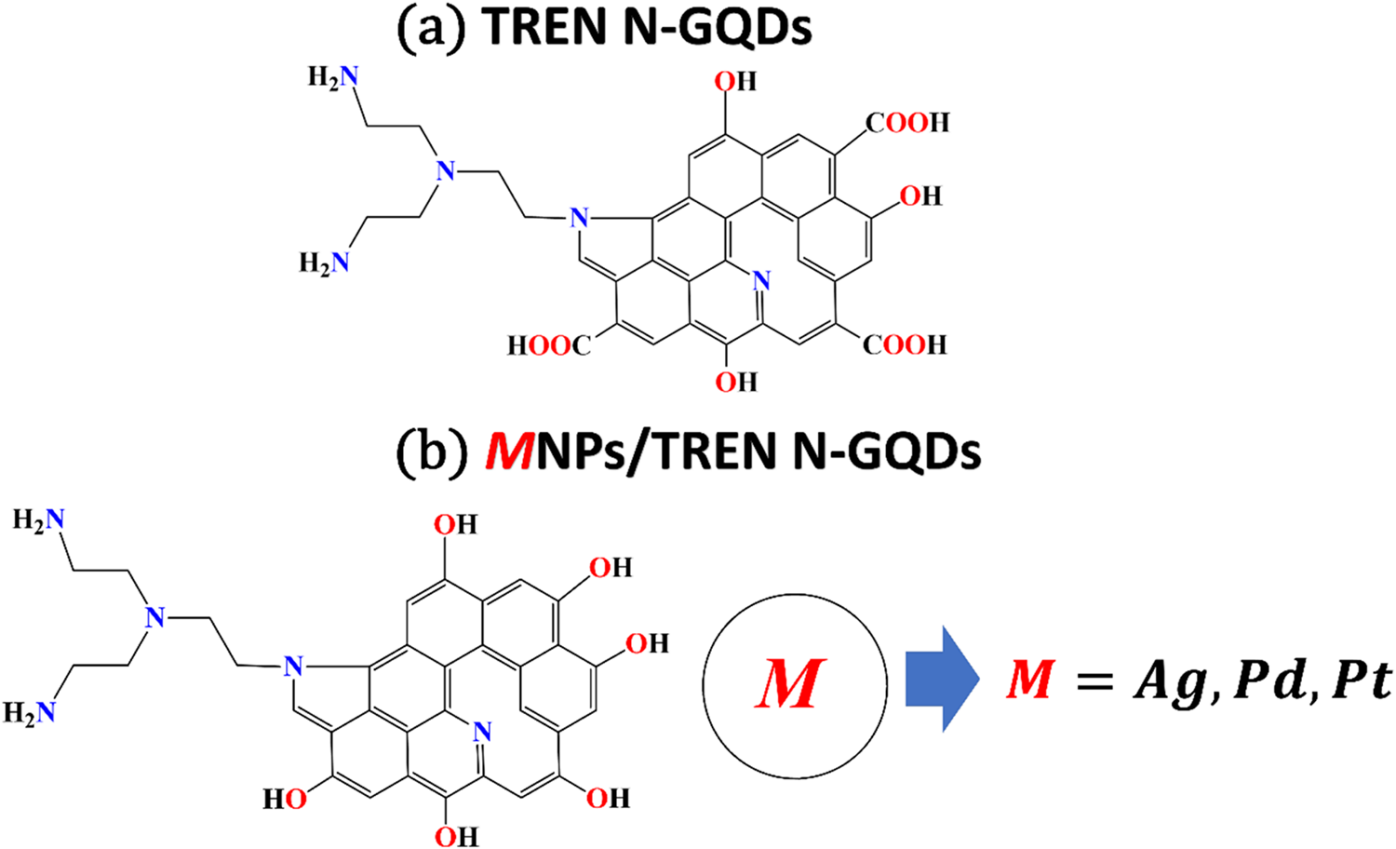
Theoretical Model of (a) TREN N-GQDs and (b) MNPs/TREN N-GQDs (M = Ag, Pd, Pt) Nanocomposites

### 2.5 Viability of Cancer Cells

In this experiment, A549 human lung adenocarcinoma cells were seeded in DMEM media at 5000 cells per well (5000/well) in a 96-well plate. The cells were treated with four separate nanocomposite TREN N-GQDs (a), AgNPs/TREN N-GQDs (b), PdNPs/TREN N- GQDs (c), and PtNPs/TREN N-GQDs (d) (designated a, b, c, and d) at doses of 25, 50, 100, and 200 μg/mL. The treatment was allowed to incubate for 4 h.

After an overnight period in fresh media, an LDH (lactate dehydrogenase) assay was conducted utilizing a Thermo Fisher Scientific kit (Thermo Scientific LDH Assay Kit, 200 assay Cat No:C20300). The LDH assay measures cell membrane integrity, indicating cell viability or cytotoxicity based on the degree of membrane damage. The results were represented as a percentage of cell viability to determine the effect of each nanocomposite concentration on the A549 *cells*.

### 2.6 *In Vitro* Imaging of TREN N-GQDs, AgNPs/TREN N-GQDs, PdNPs/TREN N- GQDs, and PtNPs/TREN N-GQDs

A549 human lung adenocarcinoma cells were planted at a density of 25,000 per well in 24-well plates. Cells were treated with the nanocomposite mentioned before, at a concentration of 100 μg/mL, and incubated for 4 h. After incubation, the cells were rinsed with DPBS to remove any remaining debris and incubated overnight in fresh media. They were then fixed with 200 μg/mL cold methanol at – 20 °C to maintain the cellular architecture and colored with DAPI to expose the nuclei. Fluorescent images were obtained with an inverted microscope (Zeiss Co.).

## 3 Results and Discussion

### 3.1 Characterization of Synthesized Materials (FT-IR, TEM, and EDX)

The FT-IR spectra of TREN N-GQDs exhibit characteristic changes in functional group frequencies when compared with the FT-IR spectra of the starting materials CA and TREN (Figure 1). C=N_pyridinic_ vibration is observed as a new peak in the newly formed TREN N-GQDs. In addition, a large number of COOH, OH, and NH_2_ vibrations as side groups are observed very strongly. Functional vibration bands in TREN N-GQDs are ν­[H_2_O+NH_2_+COOH]; 3854–3740–3230, νC–H; 2939, νC=O; 1701, νC=N_pyridinic_; 1662, νC=C; 1541, νC–N_bending_; 1437, νC–O; 1392, C–N_stretch_; 1091–1037, νO–H_bending_; 931–879, N–H_wag_; 762 and νC–H_deformation_; 625 cm^–1^, (Figure 1). In AgNPs/TREN N-GQDs nanocomposites, functional group vibrations are ν­[H_2_O+ OH+NH_2_]; 3852–3724–3392–3224, νC–H; 2935, νC=O_ketone_; 1720, νC=N _pyridinic_; 1651, νC=C; 1524, and ν­[C–N+C-O]; 1438, νC–N_stretch_; 1178, N–H_wag_; 797 and νC–H_deformation_; 618 cm^–1^ (Figure 2). The ketone (C=O) vibrations observed at 1720 cm^–1^ in Ag nanocomposites, COOH groups in TREN N-GQDs as well as OH groups, taking part in the reduction of Ag­(+), are reduced to Ag(0) while some OH are oxidized to ketone (C=O). This is not observed in other Pd and Pt nanocomposites (Figure 2).

**Figure 1:**
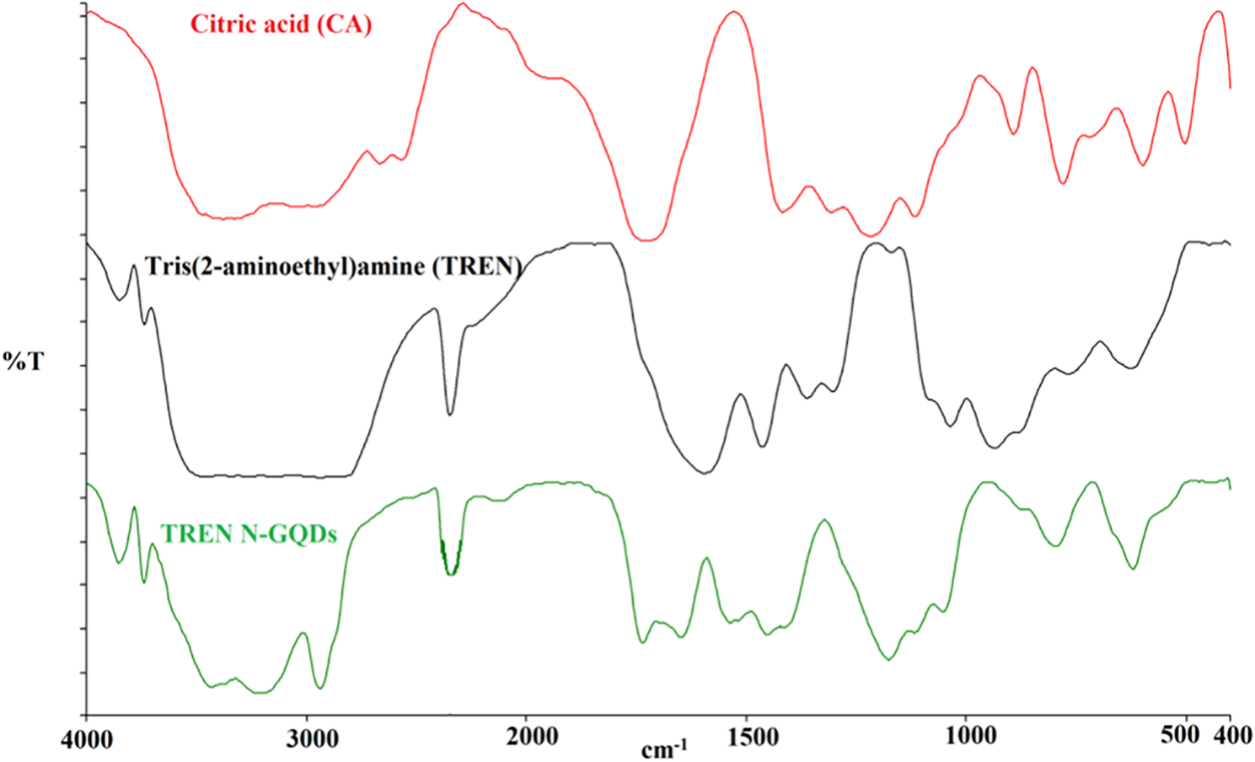
FT-IR spectra of CA, TREN, and TREN N-GQDs.

**Figure 2:**
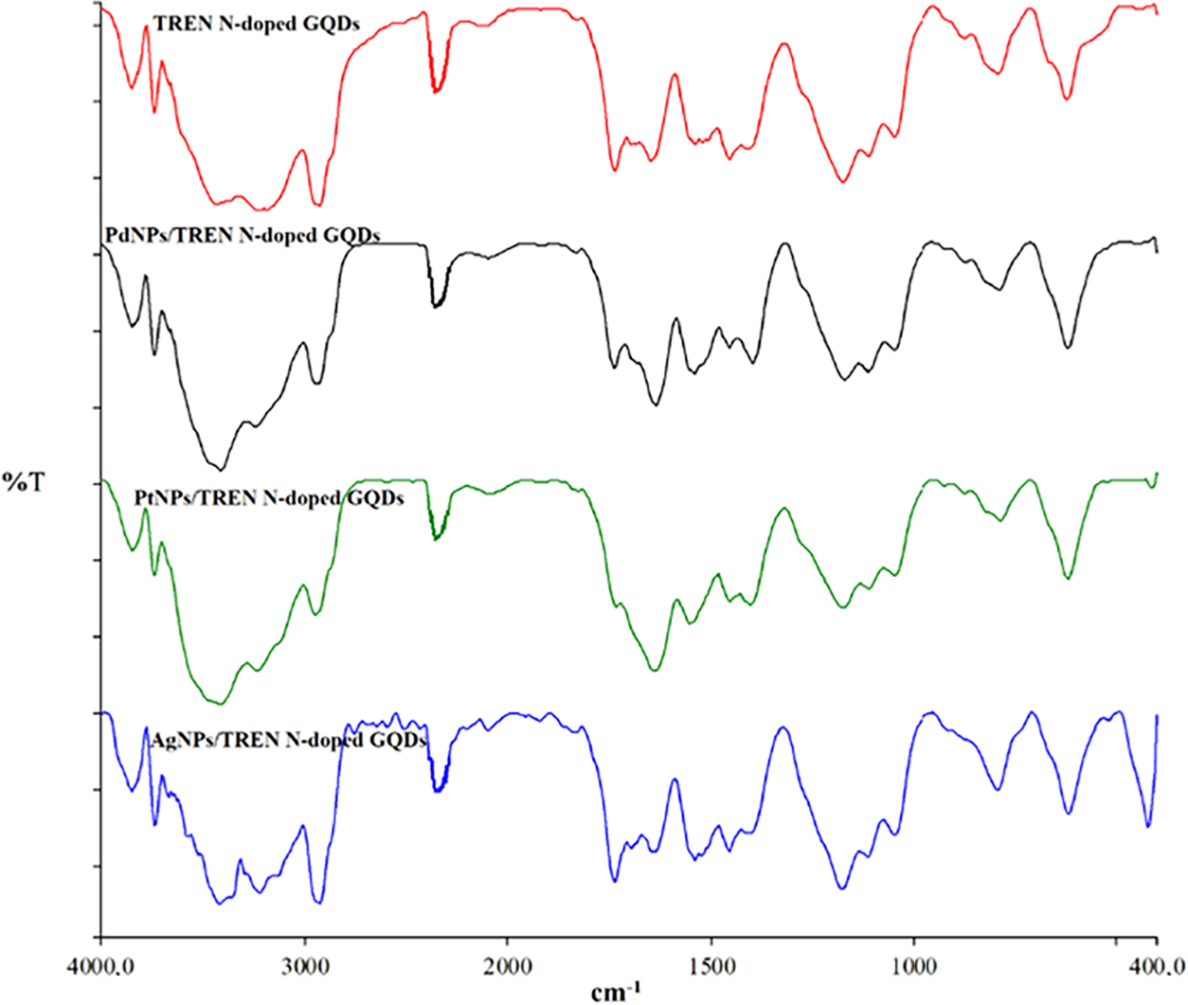
FT-IR spectra of TREN N-GQDs, AgNPs/TREN N-GQDs, PdNPs/TREN N- GQDs, and PtNPs/TREN N-GQDs.

Similar functional group vibrations were observed in Pd- and Pt-NPs/TREN N-GQDs nanocomposites, ν­[H_2_O+NH_2_+OH], νC–H, νC=N_pyridinic_, νC=C, ν­[C–N+C-O], νC–N_stretch_, N–H_wag_ and νC–H_deformation_ vibrations were observed for Pd, 3852–3724–3430–3239,2948, 1647,1539, 1419, 1159, 796, and 619 cm^–1^, and for Pt, 3852–3724–3430–3239,2948,1648, 1538, 1424, 1176, 792, and 619 cm^–1^, respectively (Figure 2).

The structure and morphology of TREN N-GQDs and Ag-, Pd-, and Pt-NPs/TREN N-GQDs nanocomposites were investigated by TEM images, as given in Figure 3. TREN N-GQDs exhibit a distorted spherical morphology with particle sizes ranging from 2 to 6 nm (Figure 3a). In AgNPs/TREN N-GQDs nanocomposites, a spherical and nonclustered structure ranging from 1–3 nm was observed (Figure 3b). This is the smallest particle size and the most spherical structure found among all of the materials. PdNPs/TREN N-GQDs nanocomposites have the highest size and the most distorted spherical structure with particle sizes of 6–30 nm among all materials (Figure 3c). The particle sizes of PtNPs/TREN N-GQDs nanocomposites were spherical, between 2 and 4 nm (Figure 3d).

**Figure 3:**
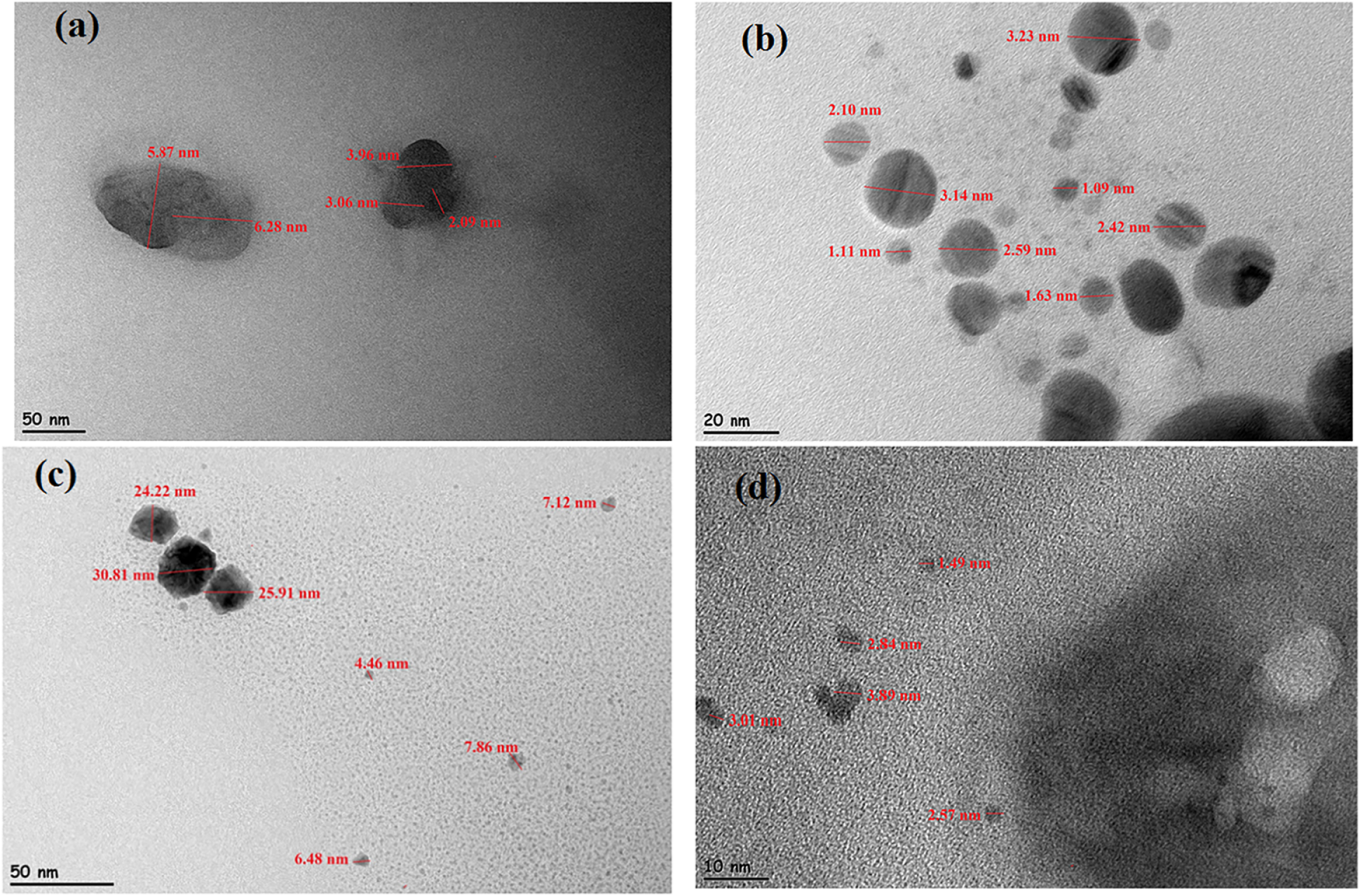
TEM analysis of TREN N-GQDs (a), AgNPs/TREN N-GQDs (b), PdNPs/TREN N- GQDs (c), and PtNPs/TREN N-GQDs (d).

The presence of elements in the materials was confirmed by scanning electron microscopy with an EDX detector. The number of elements found by the EDX method is given in Figures S1–S4. From the EDX spectrum results, carbon, nitrogen, and oxygen were detected on the surface of TREN N-GQDs. In Ag-, Pd-, and Pt-NPs/TREN N-GQDs nanocomposites, besides the elements C, N, and O, the metals Ag, Pd, and Pt were found in the measured nanocomposites (Figures S1–S4). The presence of metals on the material surface is evidence of the formation of metal nanocomposites. As is well-known, EDX only detects the content of elements on the surface and never gives a complete elemental analysis.

### 3.2 Linear Optical Properties

The absorption spectra of TREN N-GQDs and Ag-, Pd-, and Pt-NPs/TREN N-GQDs nanocomposites were measured in water (Figure 3). In the UV–vis spectrum of TREN N-GQDs, two bands assigned to π–π* transitions of C=C at 247 nm and *n*–π* transitions of C=N and C=O at 340 nm were observed (Figure 4). In TREN N-GQDs Ag, Pd, and Pt nanocomposites, π–π* and *n*–π* transitions were found at 275 and 350, 230 and 327 nm, and 271 and 348 nm, respectively. Furthermore, the shoulder at 422 and the peak at 418 nm were assigned to plasmon resonance in PdNPs/TREN N-GQDs and AgNPs/TREN N-GQDs nanocomposites (Figure 4).

**Figure 4:**
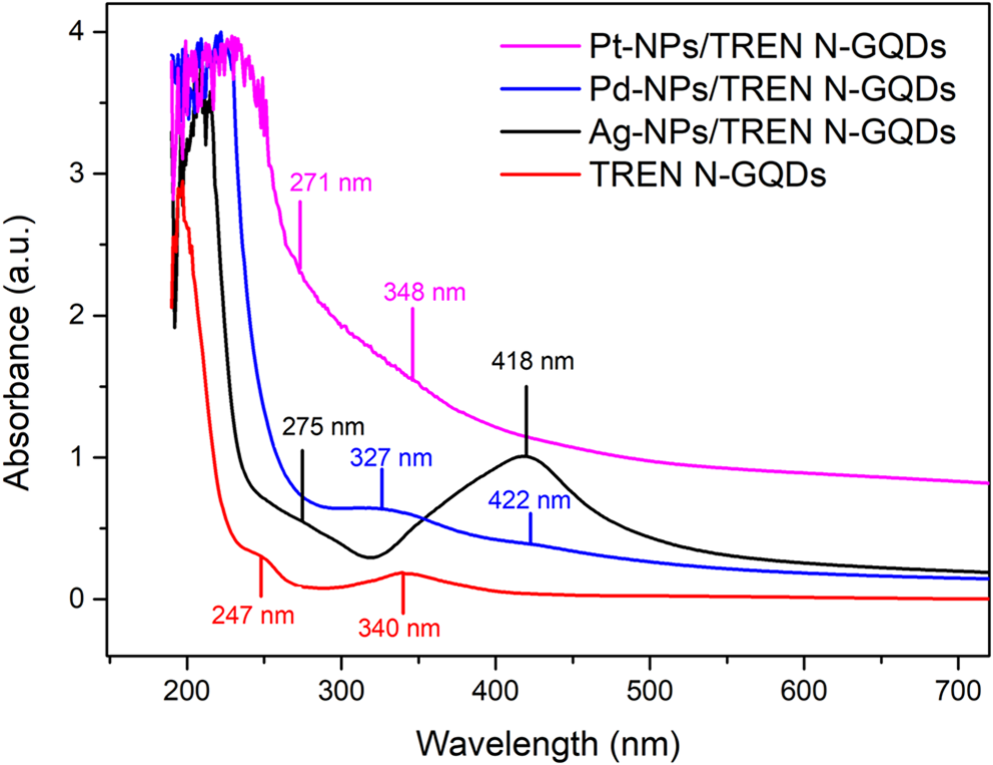
UV–vis spectra of TREN N-GQDs, AgNPs/TREN N-GQDs, PdNPs/TREN N-GQDs, and PtNPs/TREN N-GQDs.

By picturing the energy levels of the samples, we can understand the nonlinear absorption (NLA) properties more clearly. The energy level diagram provides insight into the electronic states involved, their relative energies, and possible absorption and relaxation transitions that contribute to the observed nonlinear optical behavior in Z-scan and pump–probe experiments. For this purpose, we have conducted several photoluminescence (PL) measurements to construct a representative energy level diagram and better interpret the underlying mechanisms.

Emission spectra of TREN N-GQDs with different excitation wavelengths are listed in Figure 5. Figure 5 presents the emission spectra of the TREN N-GQDs at various excitation wavelengths. Minimal shifts in emission wavelengths were observed at excitation wavelengths of 225 and 250 nm. Significant shifts in emission wavelengths occur at 275 and 300 nm excitation wavelengths. It has been seen that the emissions occurring with 325 and 350 excitation wavelengths occur at the same wavelength as the emissions resulting from 225 and 250 nm excitations, suggesting that emissions occur from the same energy state. Starting from the 375 nm excitation wavelength, the emission wavelengths shifted toward higher wavelengths.

**Figure 5:**
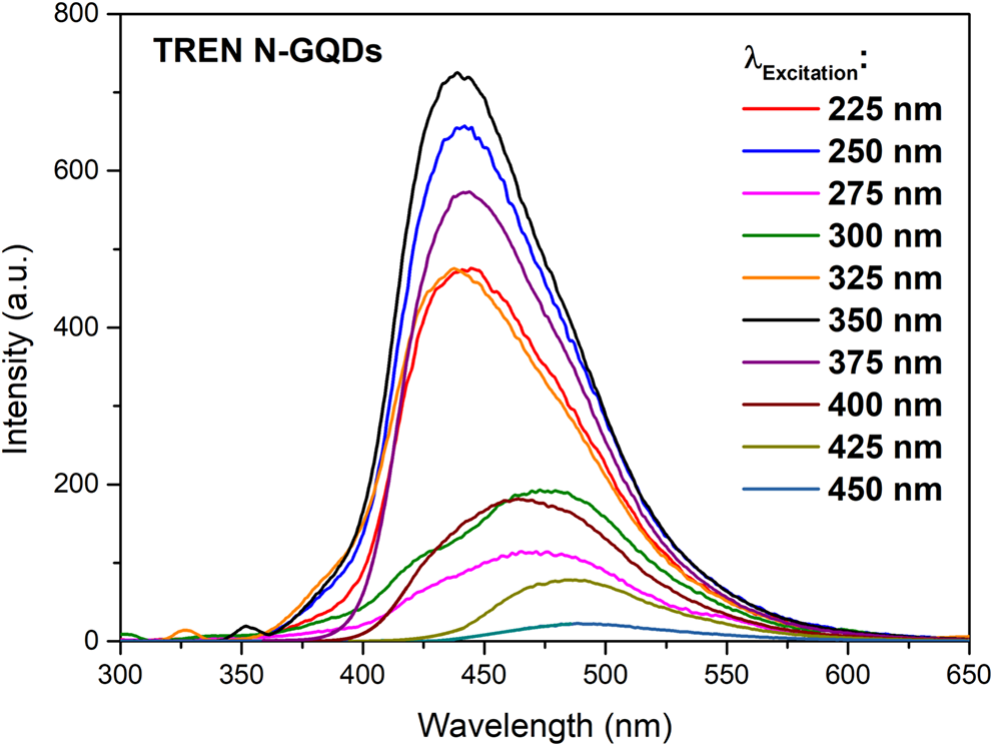
Photoluminescence emission of TREN N-GQDs at different excitation wavelengths.

To better understand the energy levels of the TREN N-GQDs structure, first, the intensity of the emission peaks versus the excitation wavelengths were plotted (Figure 6). Because of the variation of emission wavelengths with excitation wavelengths, we chose to construct the graphs by using several independent emission measurements. This approach provides more accurate spectral information than a continuous excitation–emission scan, which may not effectively capture such shifts. To reveal the underlying trend, the data are plotted as both a scatter plot and a smooth curve; however, only the scatter data are used in the calculation of the energy level diagram. The peaks observed at 250 and 352 nm suggest that the π–π* and *n*–π* transitions occur at approximately 5.00 and 3.52 eV.

**Figure 6:**
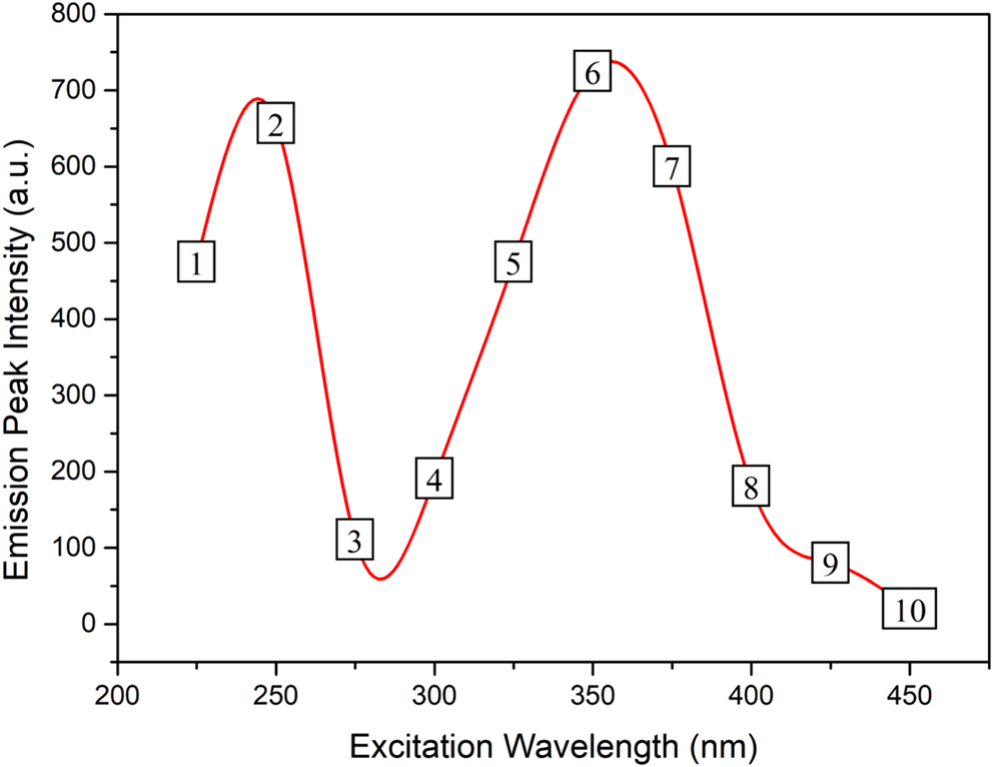
Variation of emission peak intensities with the excitation wavelength.

It is known that the excitation wavelength-dependent emission (dependent PL) in GQDs structures originates from the surface and edge defects in the structure.^48–50^ In order to depict the location of these defect levels and *n*, π, and π* energy levels, a graph of the excitation wavelength versus the emission wavelength of the TREN N-GQDs sample was drawn (Figure 7).

**Figure 7:**
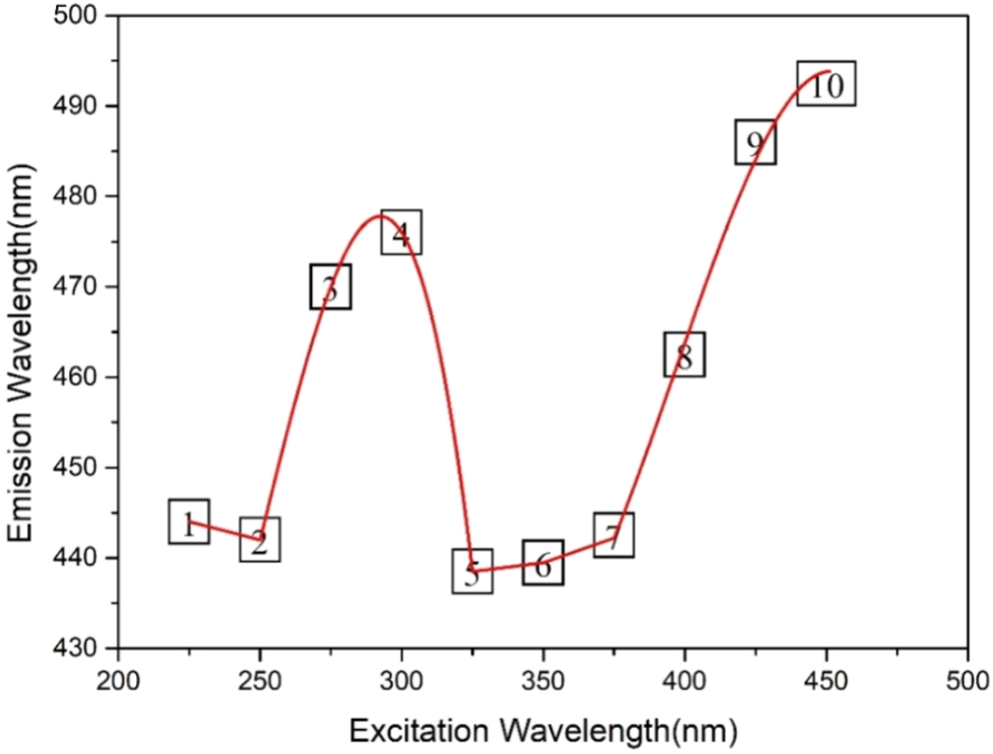
Variation of the emission wavelength with the excitation wavelength.

The energy level diagram below (Figure 8) was drawn with the calculations made from the data shown as consecutive numbers in Figure 7 above. In the explanation of the diagram, the energies corresponding to the wavelengths will be given in parentheses. And the transitions will be explained separately under the subheadings of the above-mentioned numbers.

**Figure 8:**
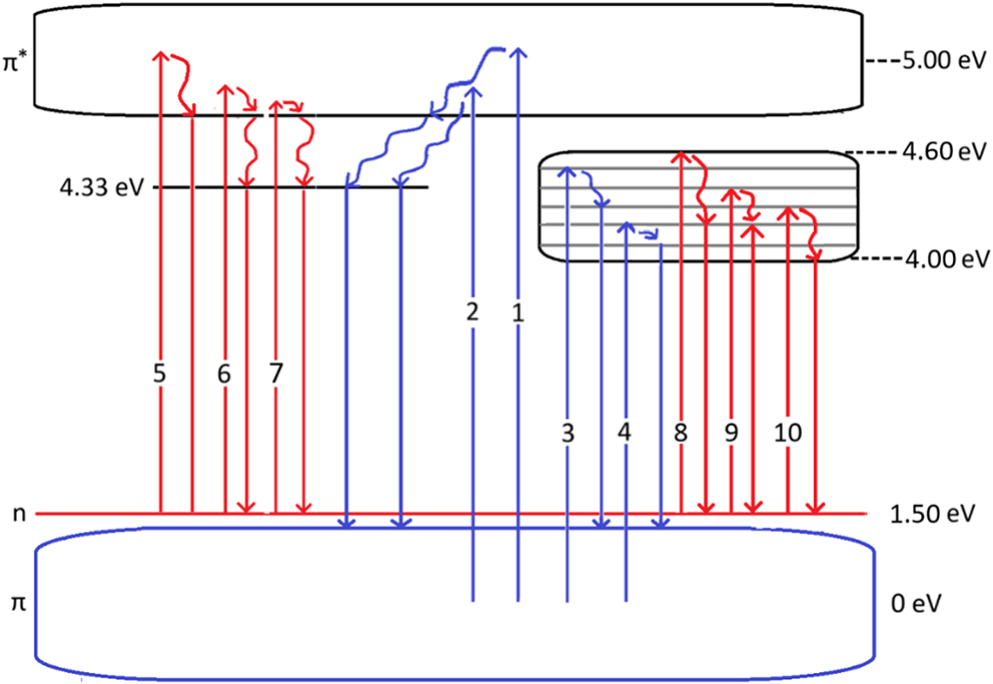
Energy level diagram of the TREN N-GQDs.

#### 3.2.1 Transition #1 and #2

At excitation wavelengths of 225 nm (5.51 eV) and 250 nm (4.96 eV), emissions were observed at nearly identical wavelengths. However, the emission energies (2.81 eV) being lower than the excitation energies and manifesting as independent photoluminescence (PL) suggest the involvement of saturated edge defect levels in the corresponding transitions. This indicates that emissions take place between saturated edge states (N states) and the edge of the p band following a nonradiative transition from π* to saturated edge state subsequent to π to π * excitation. It is established that in GQDs, the energy levels where dependent PL occurs due to edge defect levels are located closer to π* energy levels. It has also been reported in the literature that these edge defect levels can become saturated with nitrogen doping, and the emissions turn into independent PL.

#### 3.2.2 Transition #3 and #4

Upon excitation at 275 nm (4.50 eV) and 300 nm (4.13 eV) excitations, π electrons undergo a transition to the edge defect band. Subsequently, as electrons dissipate some energy via phonon levels, excitation-dependent emissions occur between the edge defect band and the π level. The fact that the emissions are dependent on PL means that the edge defect levels are partially saturated with N-doping, and the structure contains both edge defect bands containing discrete energy levels and nitrogen levels resulting from the saturation of these levels. Although not depicted in the energy level diagram, excitations at 275 and 300 nm also promote electrons from *n* levels to π* states. Following excitation, these electrons relax nonradiatively to N states and then deexcite to n states back by emitting photons, which manifest in the emission spectrum as shoulder features around 425 nm under 275 and 300 nm excitation wavelengths.

#### 3.2.3 Transition #5, #6, and #7

At excitation wavelengths of 325 nm (3.81 eV), 350 nm (3.54 eV), and 375 nm (3.31 eV), transitions between n levels and π* levels lead to emissions characterized by independent photoluminescence (PL) at a wavelength of 440 nm (2.81 eV). Analogous to the transitions observed in transitions #1 and #2, emissions stem from N states subsequent to nonradiative transitions from π* levels. However, in this case, recombinations occur at n levels instead of π levels.

#### 3.2.4 Transition #8, #9, and #10

Emissions resulting from excitation wavelengths of 400 nm (3.10 eV), 425 nm (2.92 eV), and 450 nm (2.76) predominantly exhibit dependent PL similar to #3 and #4 transitions, indicating the significant presence of edge defect states. Following excitation, electrons from the nonbonding (n) levels are promoted to the defect band. Within this band, electrons dissipate a portion of their energy nonradiatively before radiatively transiting to the n level, thus generating dependent PL.

When the emission spectra of MNPs incorporated TREN N-GQDs (Figures S5–S7) are examined, it is seen that only very negligible wavelength-dependent PL occurs at small excitation wavelengths. Furthermore, it is seen that wavelength-dependent PL occurs in a more limited range at large wavelengths, indicating the depletion of defect states. In the pump–probe experiments (to be discussed in the next section), the shortening of the lifetime of excited electrons of TREN N-GQDs when MNPs are introduced into the structure also indicates that the defect levels below the pi* band are also reduced. When the dependent PL occurring at long excitation wavelengths was examined, it was revealed that the emission originates primarily from transitions labeled as #8, #9, and #10, corresponding to electronic transitions between the defect band and localized *n* levels. The absence of wavelength-dependent PL at shorter wavelengths (transition #3 and #4) shows that MNP incorporation suppresses the PL originating from defect to pi band. This selective quenching is attributed to the intrinsic nature of the π states, which are delocalized throughout the sp^2^ carbon network and are therefore highly susceptible to perturbations from the MNPs. In particular, π states readily couple with MNPs via electronic hybridization or charge transfer, effectively blocking radiative transitions to these levels. In contrast, emission pathways involving transitions from defect states to localized *n* states remain active. These *n* levels exhibit minimal spatial overlap with the delocalized π-system and are thus less affected by MNP-induced perturbations. The persistence of defect to *n* transitions suggests that these levels are electronically and spatially isolated from the MNP-modified π system.

### 3.3 Nonlinear Absorption Properties

In order to get a deep insight into the electron transfer mechanism of the studied compounds, ultrafast pump–probe spectroscopy measurements were carried out. The pump wavelength was determined according to the wavelength corresponding to the maximum absorption value in the linear absorption spectra. In transient absorption spectra of TREN N-GQDs, there is a broad excited-state absorption (ESA) signal lying in whole spectra that has almost the same intensities at initial time delays, as seen in Figure S8. Moreover, it was observed that a small valley placed in the range of 430–530 nm and appearing 10 ps later can be attributed to stimulated emission (SE) signals competing with the ESA signals. On the other hand, the negative signal around 420 nm corresponding to ground state bleaching (GSB) was observed for AgNPs/TREN N-GQDs compound by incorporating Ag atoms, as indicated in Figure S9. The saturation of the AgNPs/TREN N-GQDs compound shows a strong interaction between Ag atoms and TREN N-GQDs. Upon photoexcitation at 300 nm of PdNPs/TREN N-GQDs compound, the GSB signal localized below 500 nm is attributed to the depletion of the ground state (Figure S10). After a few hundred fs time delay, this negative signal turns into a positive signal due to the presence of the dominant ESA signal. In the transient absorption spectra of the Pt-NPs/TREN N-GQDs compound, a broad ESA signal was observed, as seen in Figure S11.

To analyze the excited-state lifetimes of the studied compounds, the decay kinetics of the signals around 500 nm were probed, and the results are presented in Figure 9. Based on the decay traces, it is clearly seen that the lifetime is shortened by incorporating the metals into the TREN N-GQDs compound.

**Figure 9:**
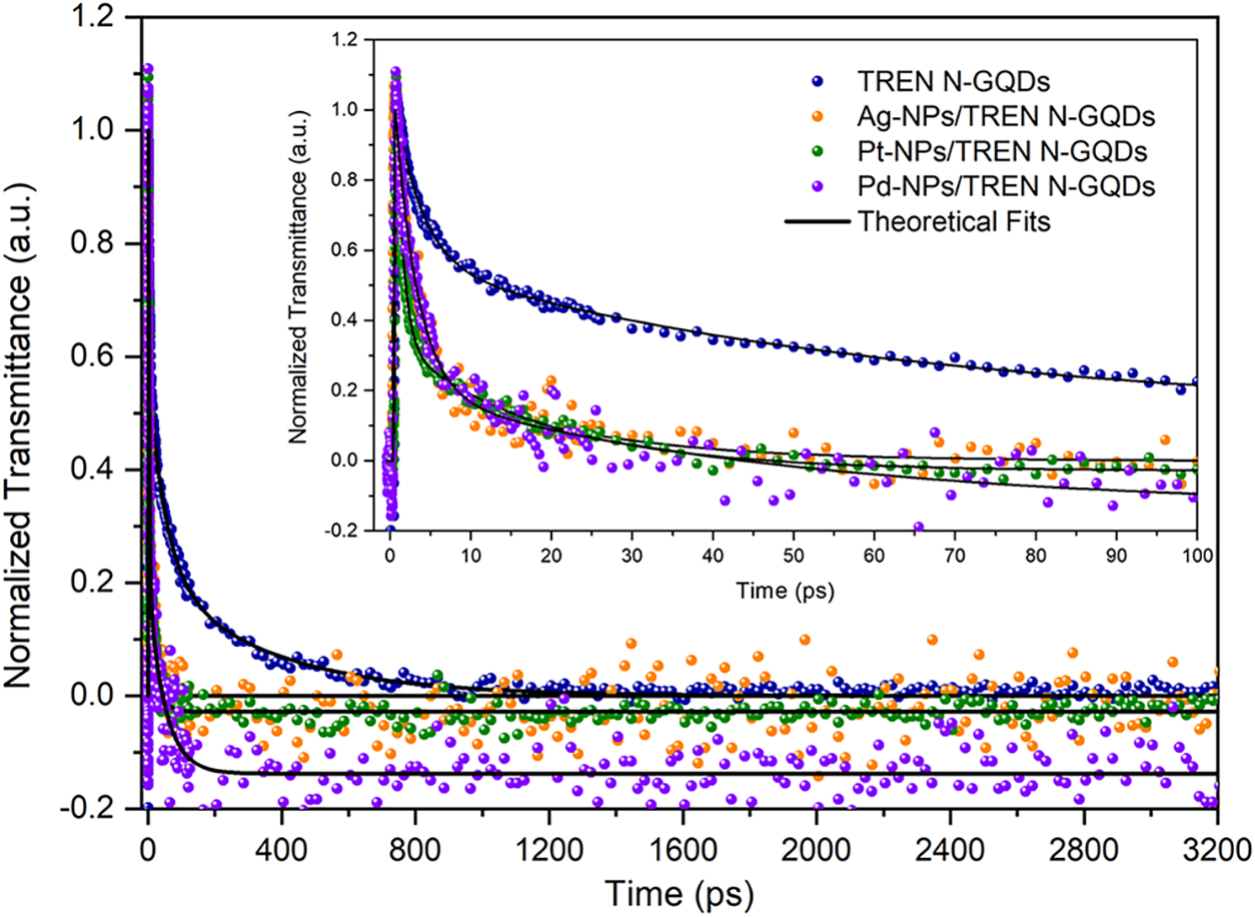
Decay kinetics of TREN N-GQDs and nanocomposites.

The emission occurring over a wide wavelength range observed in fluorescence measurements suggests that there is a non-negligible amount of defect states in the band gap of the GQDS samples. Considering these defect states, it is necessary to evaluate the nonlinear absorption behavior not only in the TPA mechanism but also in one-photon absorption (OPA) and excited-state absorption (ESA) mechanisms. Therefore, in the evaluation of OA Z-scan experiments, models including OPA, TPA, ESA, and saturation of each absorption mechanism were considered.^51–54^

1 $$\frac{dI}{dz}=-\frac{\alpha I}{1+I/I_{\mathrm{sat}}}-\frac{\beta I^{2}}{1+I^{2}/I_{\mathrm{sat}}^{2}}-\frac{\sigma_{o}\Delta N\left(I\right)I}{1+I^{2}/I_{\mathrm{sat}}^{2}}$$

In eq 1, each term corresponds sequentially to the numbers of OPA, TPA, and ESA. σ_o_ represents the ESA cross section, while α and β denote the OPA and TPA coefficient, respectively. The photoexcited free carrier density, Δ*N*(*I*), varies with intensity, and *I*_sat_ refers to the saturation intensity threshold.

The photoexcited free carrier density can be expressed as a function of α and β as^55,56^

2 $$\Delta N=\frac{\alpha I}{\hbar \omega }\tau_{0}+\frac{\beta I^{2}}{2\hbar \omega }\tau_{0}$$

Here, τ_0_ represents the pulse duration, and ℏω denotes the photon energy. By substitution of eq 2 into eq 1, the following equation can be obtained.

3 $$\frac{dI}{dz}=-\frac{\alpha I}{1+I/I_{\mathrm{sat}}}-\frac{\beta_{\mathrm{eff}}I^{2}}{1+I^{2}/I_{\mathrm{sat}}^{2}}$$

The term β_eff_ in the above equation represents the effective NLA coefficient, incorporating the contribution from both ESA and TPA, and was given by $\beta_{\mathrm{eff}}=\beta +\left[\frac{\sigma_{o}\tau_{0}}{\hbar \omega }\left(\alpha +\frac{\beta I}{2}\right)\right]$ .

The Adomian decomposition method was employed to solve eq 3 and was used to fit the experimental OA Z-scan curves.^52,53^

Nonlinear absorption responses of MNPs*/*TREN N-doped GQDs samples are given in Figure 10. Experiments were carried out at a wavelength of 800 nm, a pulse duration of 1 ps, and a laser intensity of 70 GW/cm^2^. All of the samples showed absorption behavior, but no dominant saturable absorption was observed. The disappearance of wavelength-dependent emission at small wavelengths with the addition of Pt and Pd to the structure indicates that these nanoparticles saturate the edge defect levels. The depletion of the edge defect levels leads to shorter lifetimes—from the nanosecond range in TREN N-GQDs to 40–50 ps with the incorporation of MNPs, as revealed by the decay kinetics, and enhances nonlinear absorption (NLA) by allowing electrons to be re-excited upon returning to the ground state by two-photon absorption. In other words, this reduces the negative impact of saturable absorption on NLA. While a similar mechanism occurs with the addition of Ag to the structure, the nonlinear absorption response was further strengthened in TREN-Ag samples by excitation of surface plasmons with two-photon absorption (at the wavelength corresponding to a 400 nm wavelength). The excitation at half of the SPR wavelength and the absence of defect states at 800 nm wavelength mean there are no suitable intermediate energy levels corresponding to the absorption of the first photon. Therefore, the sequential two-photon absorption pathway is effectively suppressed. Under these conditions, the observed nonlinear absorption is most likely due to simultaneous two-photon absorption occurring through the surface plasmon resonance (SPR) absorption wavelength, where two photons combine their energies to excite the system directly via the plasmonic states.^57–60^ In addition, two-photon absorption occurs simultaneously for Pt and Pd incorporated N-GQDs; however, the absence of pronounced SPR, caused by strong interband damping, limits the transitions to the surface states only around the wavelength of 400 nm, resulting in a lower NLA output compared to AgNPs*/*TREN N-doped GQDs. In conclusion, NLA is further strengthened by the additional contribution of Ag’s plasmon absorption band via simultaneous two-photon absorption, revealing the highest absorption in z-scan traces. βeff values of 1.30 × 10^–9^ m/W, 2.11 × 10^–9^ m/W, 2.35 × 10^–9^ m/W, and 3.56 × 10^–9^ m/W for TREN N-doped GQDs, Pt-, Pd- and AgNPs/TREN N-doped GQDs samples have been found, respectively. The intensity values (*I*_sat_), at which the saturable absorption mechanism occurs where the absorption properties of the materials decrease at high laser intensities and, in extreme cases, behave in such a way that the normalized transmittance exceeds one, were also obtained as a result of the refinement. *I*_sat_ values were found to be 478, 629, 705, and 815 GW/cm^2^, respectively.

**Figure 10:**
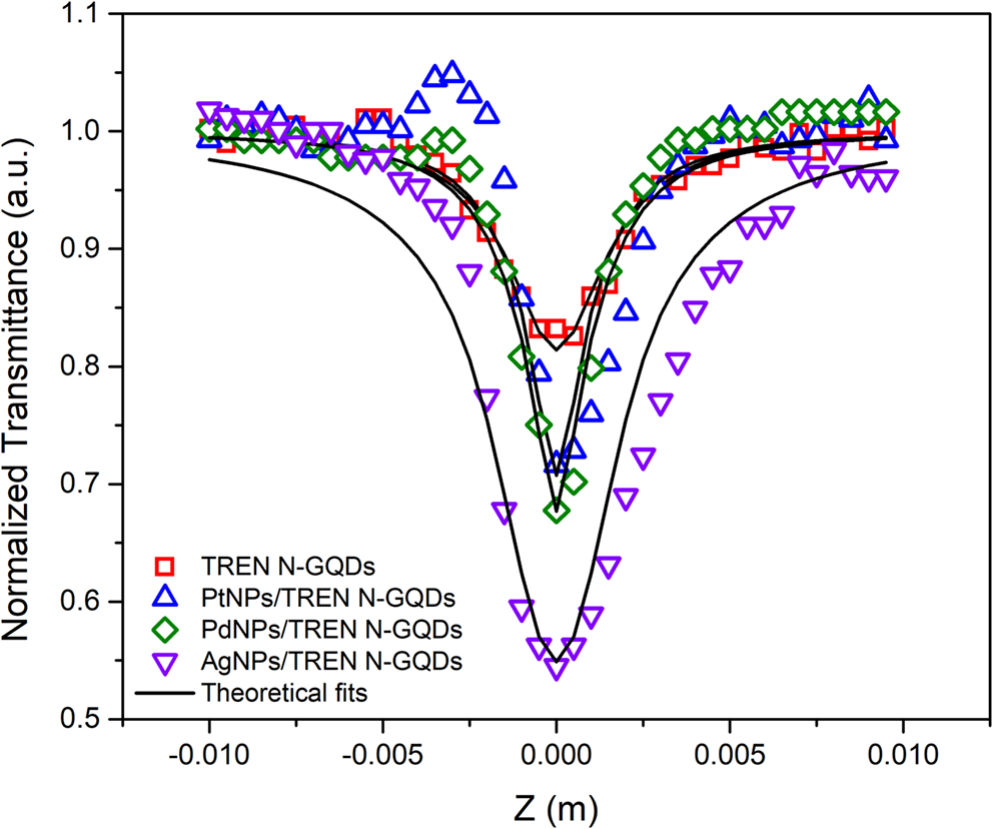
Z-scan traces of TREN-functional N-doped GQDs/MNPs samples at 800 nm wavelength and 70 GW/cm^2^ intensity.

### 3.4 DFT Analysis

The electronic structures of TREN N-GQDs and MNPs/TREN N-GQDs (M = Ag, Pd, and Pt) nanocomposites were thoroughly examined, and their properties were predicted by optimizing them using density functional theory (DFT).^61,62^ By minimizing the total energy of the system, DFT allows us to determine the most stable configurations, offering insights into preferred binding geometries and electronic interactions. An understanding of the reactivity of the nanocomposite and its interactions with biological molecules like DNA depends on the identification of critical properties such as energy gaps, charge distribution, and molecular orbitals, which are exposed by this optimization. This theoretical approach provides a thorough framework for atomic-level optimization of the nanocomposite’s structure and properties, which improves functionality and efficiency in the intended applications. First, the lowest energy structure representing the most stable configuration of the nanocomposites was calculated through geometrical optimization in the gas phase. This optimization in the gas phase improves our understanding of the fundamental properties and behavior of the materials we are studying by reducing external influences and permitting molecules to move freely. With the help of this technique, it is possible to observe the basic energy levels of molecular structures in a situation in which there are few intermolecular interactions, and molecules are free to move around. The gas phase, therefore, offers an ideal environment to explore the natural structures and fundamental energy levels of the molecules. But it is crucial to analyze the materials under study in aqueous media in order to reconcile quantum chemical computations with experimental findings. This is because experimental studies are usually carried out in solvent media, and calculations must be performed in this environment to accurately reflect the real-world behavior of materials. For this reason, following the gas-phase optimizations, calculations were also performed using a solvent medium similar to the experimental studies, so that the behavior of the materials was also evaluated in detail in aqueous media. As shown in Figure 11, the energy levels of nanocomposites are important indicators of structural stability. The energy levels obtained in the gas phase for the investigated nanocomposites are −2339.697, −2145.357, −2126.520, and −2118.923 hartree, respectively. These optimized configurations are a testament to the strong stability of the material structures, and in some cases, this arrangement represents the most stable structure possible. This stability enables nanocomposites to exhibit robustness and reliability in real-world applications across a variety of environments. Notably, PtNPs*/*TREN N-GQDs nanocomposite stands out by exhibiting a lower energy level compared to other materials. This low energy level underscores the superior stability of PtNPs*/*TREN N-GQDs, suggesting that this material could offer potential benefits such as increased binding affinity in applications like drug development.

**Figure 11:**
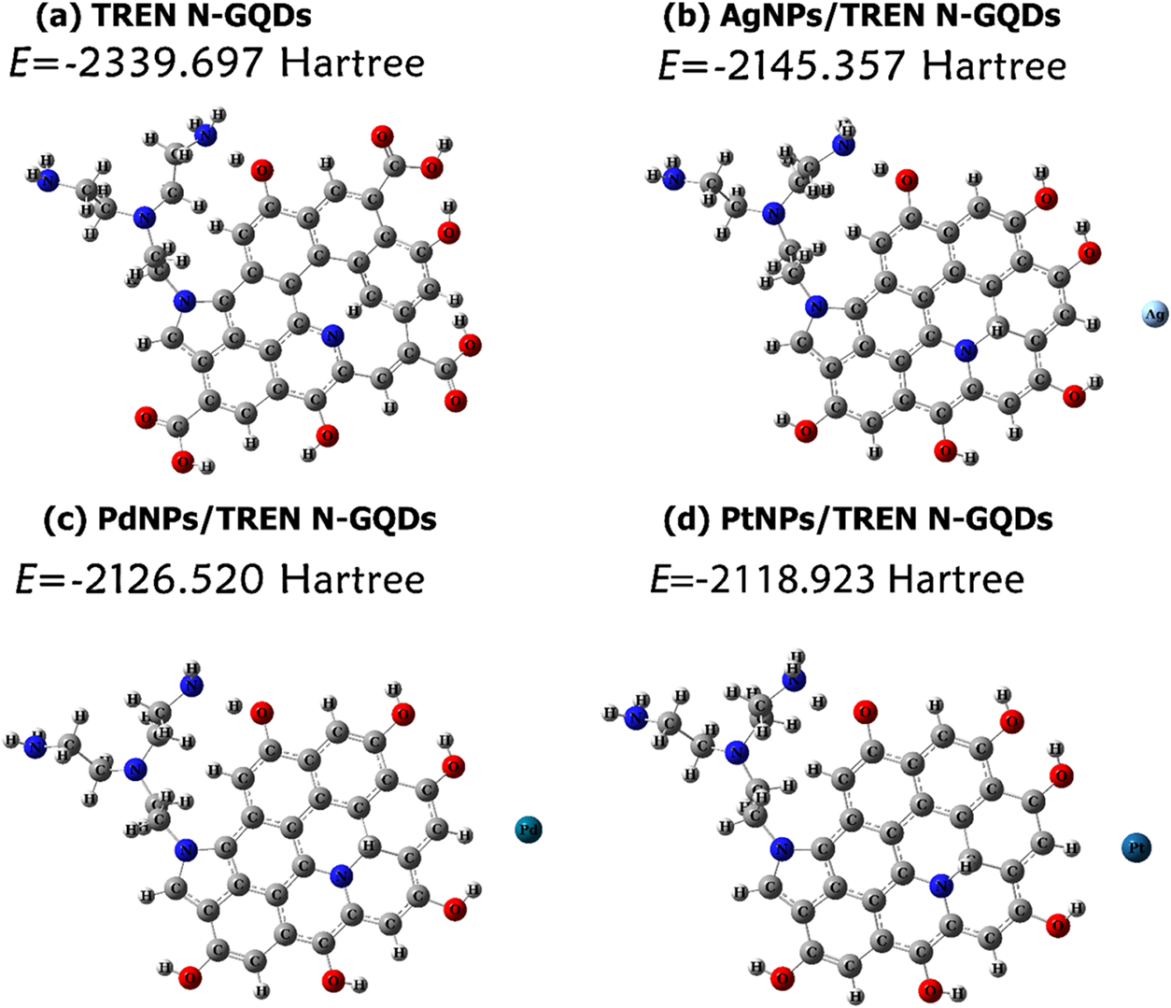
Optimized structures of (a) TREN N-GQDs, (b) AgNPs/TREN N-GQDs, (c) PdNPs/TREN N- GQDs, and (d) PtNPs/TREN N-GQDs nanocomposites.

Frontier molecular orbitals (FMO) analysis is a key concept in molecular orbital theory that plays a crucial role in understanding the chemical reactivity, stability, and various electronic properties of molecules. This analysis focuses on the highest occupied molecular orbital (HOMO), which donates electrons, and the lowest unoccupied molecular orbital (LUMO), which accepts electrons. The energy difference between HOMO and LUMO, known as the HOMO–LUMO gap, determines the reactivity and stability of a given molecule, with a smaller gap indicating greater reactivity and a larger gap indicating greater stability. FMO analysis is important for predicting chemical behavior, designing molecules with specific electronic properties, and using computational methods such as DFT for detailed insights. It has significant applications in drug design, materials science, and catalysis, making it an essential tool in theoretical and computational chemistry for the advancement of various scientific fields. Figure 12 shows the energy gaps, HOMOs, and LUMOs of the nanocomposites. The HOMO–LUMO energy gap is the smallest in the AgNPs/TREN N-GQDs nanocomposite, suggesting a lower energy needed for the electron transition from HOMO to LUMO. Because they can absorb light in the visible or near-visible spectrum and generally show higher chemical reactivity due to easier electron transitions, compounds with narrow HOMO–LUMO gaps are valuable for photovoltaic applications or as sensitizers in photochemical processes.^63^ When the energy difference between these two orbitals is minimal, electrons can be excited to higher energy states with less input energy. This reduced energy demand for electronic transitions leads to lower excitation energies, enabling easier access to multiple excited states. The interaction between the metal atoms (Ag, Pd, or Pt) and the surrounding ligands in the nanocomposites contributes to this reduced energy gap by altering the electronic structure, further facilitating these lower-energy transitions. Additionally, the charge density distribution of the nanocomposites shows delocalization in the red and green regions on the HOMO–LUMO isosurfaces, representing positive and negative charges while remaining localized elsewhere. LUMO indicates electron acceptance, while HOMO represents electron donation. Higher HOMO energy levels promote electron donation, while lower LUMO energy levels promote electron acceptance.

**Figure 12:**
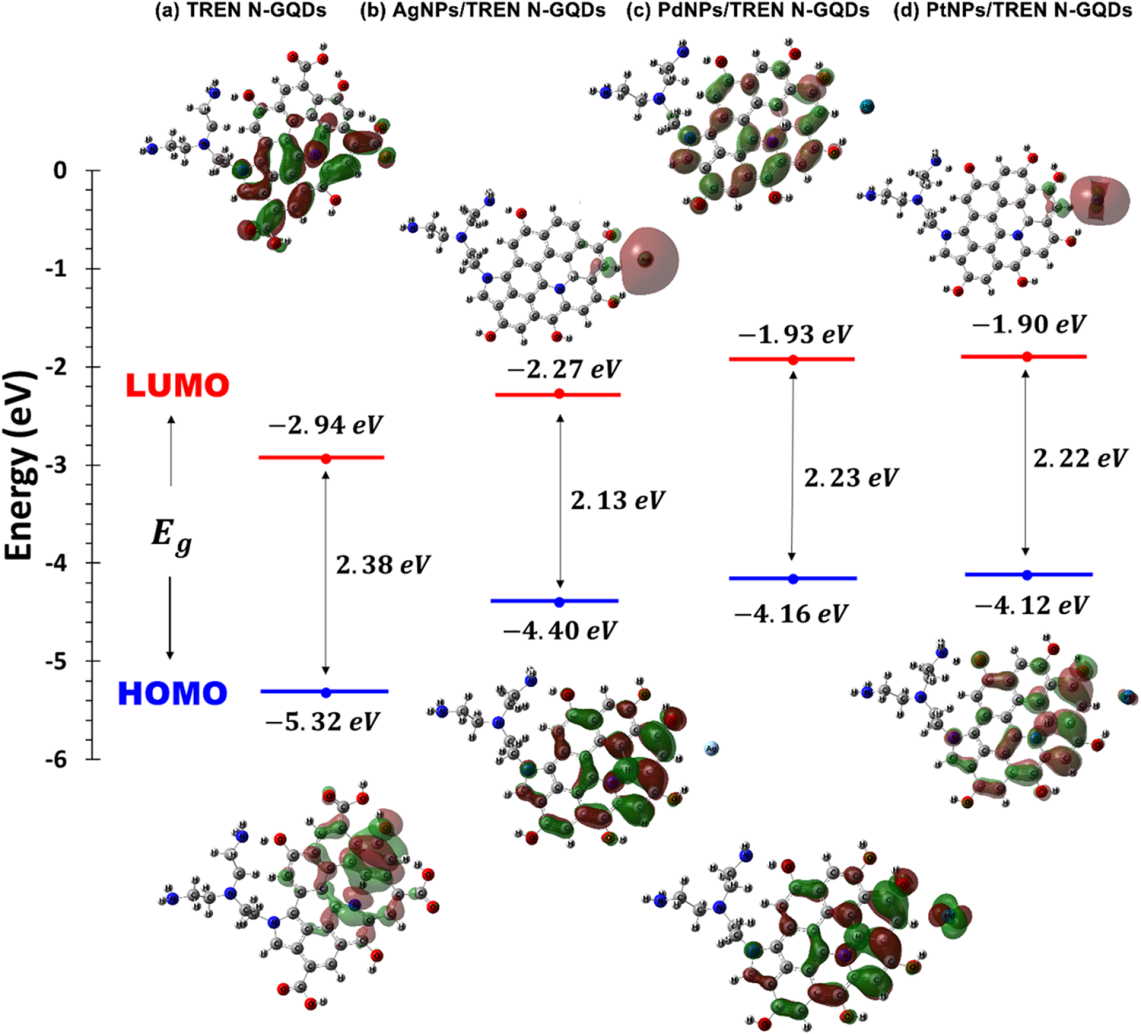
FMOs energies of (a) TREN N-GQDs, (b) AgNPs/TREN N-GQDs, (c) PdNPs/TREN N- GQDs, and (d) PtNPs/TREN N-GQDs nanocomposites.

Additionally, the charge density distribution of the nanocomposites shows delocalization in the red and green regions on the HOMO–LUMO isosurfaces, representing positive and negative charges while remaining localized elsewhere. The ability of a molecule to accept electrons is indicated by the LUMO, whereas the ability to donate electrons is indicated by the HOMO. Higher HOMO energy levels promote electron donation, while lower LUMO energy levels promote electron acceptance.

The HOMO of TREN N-GQDs is primarily localized on the nitrogen and graphene quantum dot structure. This indicates that the highest-energy electrons are delocalized over the nitrogen-functionalized sites and the aromatic system of the GQDs. The LUMO is usually delocalized on the graphene quantum dot, indicating that the addition of electrons will take place in these regions. The HOMO–LUMO gap is relatively large, indicating a low reactivity in terms of electron transfer processes.

The addition of Ag nanoparticles usually raises the HOMO energy due to the metallic nature of Ag, resulting in increased electron density around the metal-GQD interface. The LUMO extends over the silver atom and adjacent OH and CH, indicating the potential for electron transfer and the catalytic activity of the complex. The HOMO–LUMO gap is much smaller compared to TREN N-GQDs alone, indicating increased reactivity due to the metal’s ability to accept electrons. This makes the composite more suitable for applications such as catalysis and sensing. Pd nanoparticles significantly affect HOMO due to their catalytic properties. The electron density is localized on the graphene quantum dot structure and Pd. The LUMO is again delocalized on the graphene quantum dot structure and partially on Pd. Pd nanoparticles can facilitate electron transfer processes by reducing the LUMO energy. The energy gap for PdNPs/TREN N-GQDs is smaller compared to that for TREN N-GQDs, indicating high reactivity and potential catalytic activity. Pt nanoparticles will raise the HOMO energy due to their strong metallic character. The interaction with TREN N-GQDs causes a significant electron density around the Pt-TREN N-GQDs interface. Due to the lower energy of the LUMO, this promotes efficient acceptance of electrons. Pt nanoparticles significantly reduce the HOMO–LUMO gap, enhancing the reactivity of the composite and making it highly suitable for catalytic and electronic applications.

To gain a deeper insight into the sensing mechanisms of graphene quantum dots (GQDs), it is essential to analyze their UV–vis spectra.^64,65^ The excitation of electrons, which manifests as UV–vis spectra, plays a pivotal role in understanding the sensing capabilities of GQDs. To complement the experimental data for the investigated nanocomposites, electronic transitions in water were calculated across the UV–vis spectrum, focusing on the wavelengths with the highest absorption values. This analysis yielded information on absorption wavelengths, excitation energies, and the predominant electronic transitions, particularly emphasizing those with the highest oscillator strengths, as detailed in references 66,67 . Table 1 provides a comparison of the optical properties of TREN N-GQDs and their composites with silver (AgNPs), palladium (PdNPs), and platinum (PtNPs) nanoparticles. It includes both experimental and calculated values for λ_max_, oscillator strength, and excitation energy as well as the major electronic transitions.

**Table 1:** Theoretical and Experimental UV–visible Analyses of the Investigated Nanocomposites

**nanocomposites**	**experimental** **λ**_**max**_ **(nm)**	**calculated** **λ**_**max**_ **(nm)**	**oscillator strength (au)**	**excitation energy (eV)**	**major contributions**
**TREN N-GQDs**	340	344	0.16	3.6	H-6 → LUMO (76%)
					H-13 → L+2 (11%)
	247	238	0.15	5.2	H-12 → L+1 (10%)
					H-5 → L+4 (14%)
					H-1 → L+8 (34%)
**AgNPs/TREN N-GQDs**	418	440	0.59	2.82	H-1 → L+1 (91%)
					H-3 → L+2 (66%)
	360	360	0.36	3.44	H-2 → L+2 (10%)
					H-1 → L+3 (13%)
	275	273	0.21	4.54	HOMO → L+10 (38%)
					H-3 → L+4 (12%)
**PdNPs/TREN N-GQDs**	422	439	0.54	2.82	H-6 → LUMO (90%)
					H-9 → L+1 (13%)
	327	332	0.5	3.74	H-8 → L+2 (13%)
					H-6 → L+3 (49%)
					H-17 → LUMO (34%)
	230	226	0.12	5.49	H-6 → L+9 (19%)
					HOMO → L+12 (11%)
**PtNPs/TREN N-GQDs**	348	346	0.51	3.58	H-1 → L+4 (12%)
					H-1 → L+6 (54%)
	271	274	0.18	4.53	H-13 → L+1 (42%)
					H-7 → L+4 (39%)

For the TREN N-GQDs nanocomposite, the experimental λmax values are 340 and 247 nm, with calculated values closely matching at 344 and 238 nm, respectively. The oscillator strengths are relatively low, at 0.16 and 0.15 au. The excitation energies are 3.60 and 5.20 eV. Major electronic transitions include H-6 to LUMO (76%) at 340 nm and multiple contributions at 247 nm, such as H-13 to L+2 (11%), H-12 to L+1 (10%), H-5 to L+4 (14%), and H-1 to L+8 (34%).

For the AgNPs/TREN N-GQDs nanocomposite, the addition of silver nanoparticles causes a red shift, with experimental λmax values at 418, 360, and 275 nm, and calculated values at 440, 360, and 273 nm. The oscillator strengths increase significantly to 0.59, 0.36, and 0.21 au. The excitation energies are lower, at 2.82, 3.44, and 4.54 eV, reflecting enhanced optical activity. Major electronic transitions include a dominant H-1 to L+1 (91%) at 418 nm, H-3 to L+2 (66%) at 360 nm, and HOMO to L+10 (38%) at 275 nm, indicating strong interactions between the N-GQDs and silver nanoparticles.

For PdNPs/TREN N-GQDs, with the addition of palladium nanoparticles, the experimental λmax values are 422, 327, and 230 nm, closely aligning with the calculated values of 439, 332, and 226 nm. The oscillator strengths are relatively high, at 0.54, 0.50, and 0.12 au. The excitation energies are 2.82, 3.74, and 5.49 eV. Major electronic transitions include H-6 to LUMO (90%) at 422 nm, H-9 to L+1 (13%) at 327 nm, and H-17 to LUMO (34%) at 230 nm, indicating complex interactions with the palladium nanoparticles.

For PtNPs/TREN N-GQDs, the experimental λmax values are 348 and 271 nm, with calculated values closely matching at 346 and 274 nm. The oscillator strengths are 0.51 and 0.18 au. The excitation energies are 3.58 and 4.53 eV. Major electronic transitions include H-1 to L+4 (12%) and H-1 to L+6 (54%) at 348 nm and H-13 to L+1 (42%) and H-7 to L+4 (39%) at 271 nm.

The incorporation of Ag, Pd, and Pt nanoparticles into TREN N-GQDs markedly improved the optical properties of the nanocomposites. The λ_max_ values exhibit red shifts, the oscillator strengths increase, and the excitation energies change, indicating stronger absorption/emission characteristics and more intricate electronic interactions. These enhancements suggest that metal nanoparticle composites with TREN N-GQDs have greater potential for applications in sensing, imaging, and phototherapy due to their improved optical properties.

### 3.5 *In Vitro* Imaging and Cell Viability

To evaluate the in vitro imaging potential of four nanocomposites—(a) TREN N-GQDs, (b) AgNPs/TREN N-GQDs, (c) PdNPs/TREN N-GQDs, and (d) PtNPs/TREN N-GQDs—A549 lung cancer cells were incubated for 4 h with each. Following the treatment duration, the cells were examined under a confocal microscope, and representative images are shown in Figure 13.

**Figure 13:**
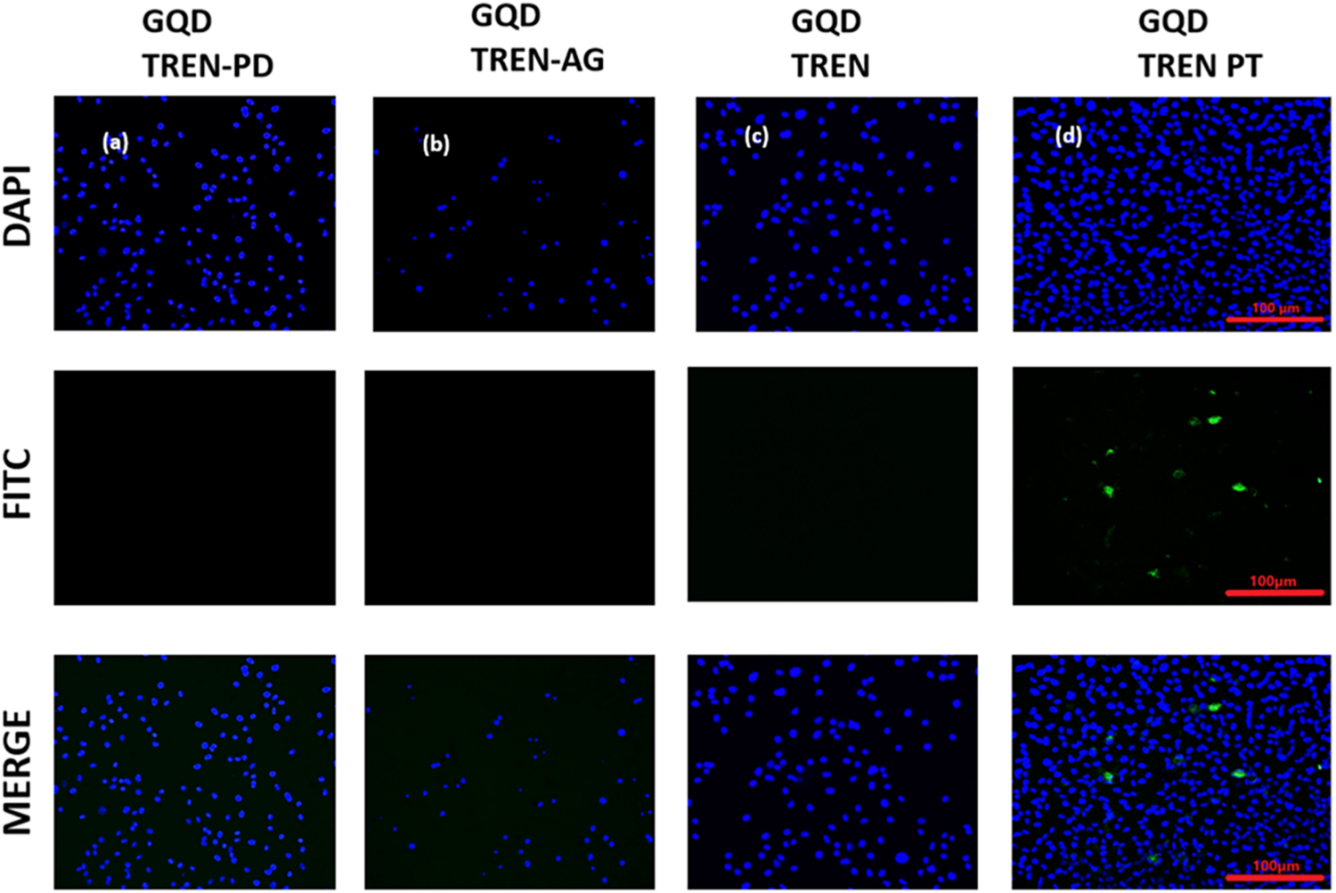
In vitro imaging of (a) PdNPs/TREN N-GQDs, (b) AgNPs/TREN N-GQDs, (c) TREN N-GQDs, and (d) PtNPs/TREN N-GQDs nanocomposites. The A549 cells were exposed to 4 h at a concentration of 100 μg/mL treatment with different materials. The cells were visualized using a confocal microscope at a magnification of 20×. DAPI was employed as a counter-stain for the nuclei. Scale bar: 100 μm.

A549 lung cancer cells were labeled with DAPI (4′,6-diamidino-2-phenylindole), a blue-fluorescent DNA dye that increases fluorescence when attached to AT sections of dsDNA. It is triggered by a violet laser (405 nm) and is commonly used as a nuclear stain in fluorescence microscopy. FITC (fluorescein isothiocyanate) is a derivative of fluorescein stain, and FITC’s excitation and emission spectra peak wavelengths are roughly 495 and 519 nm, respectively. FITC channel of the microscopes can be used to visualize the fluorescence of fluorescent molecules in the same range; therefore, the FITC channel is used to refer to the fluorescence of the GQDs.

Intracellular fluorescence was seen only in PtNPs/TREN N-GQDs-treated cells after the 4 h treatment window. After imaging, cell viability was measured 24 h later with an LDH assay.

As demonstrated in Figure 14, treatment with nanocomposites (a), (b), and (c) had a significant effect on A549 cell viability. It can be seen that (b) nanocomposite showed strong cytotoxicity even at low concentrations, resulting in cancer cell death. On the other hand, (d) nanocomposite had no significant effect on cell viability, which can be considered as a biocompatible nanocomposite for bioimaging.

**Figure 14:**
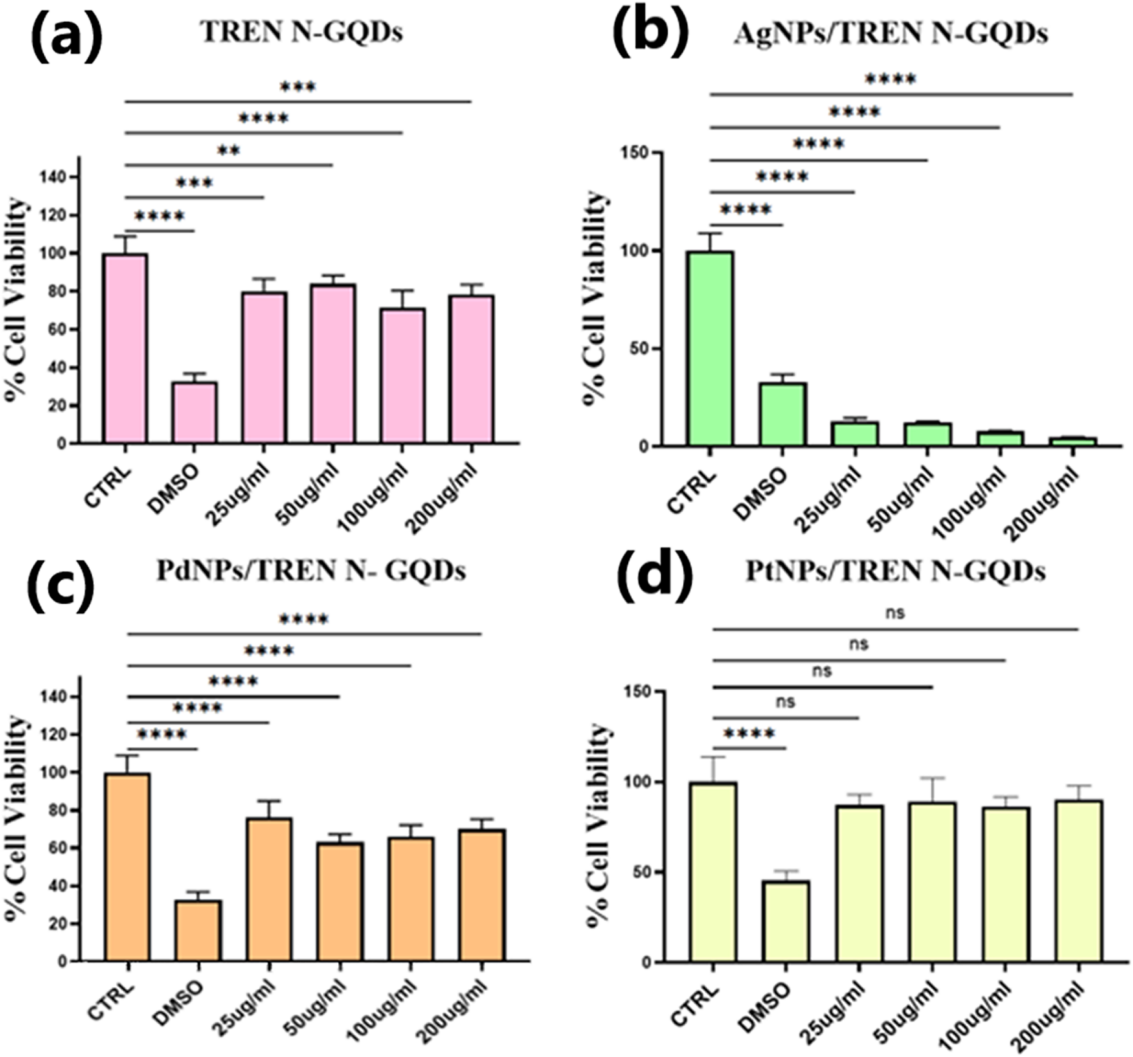
Cell viability statistics for A549 cells treated with (a) TREN N-GQDs, (b) AgNPs/TREN N-GQDs, (c) PdNPs/TREN N-GQDs, and (d) PtNPs/TREN N-GQDs nanocomposites. The cells were exposed to each substance for 4 h, and their vitality was evaluated 24 h afterward with the LDH test. The cytotoxic effects of each nanocomposite on A549 cells were analyzed with the one-way ANOVA. ((ns): nonsignificant, (**): *p*= 0.0021, (***): *p*= 0.0002, (****): *p*< 0.0001).

The use of GQDs in bioimaging demands materials with minimal cytotoxicity to ensure accurate, reproducible, and clinically relevant outcomes. Low-toxicity nanomaterials are less likely to disrupt cellular homeostasis, alter gene expression, or induce inflammatory responses, which could otherwise confound imaging results or harm biological systems during repeated imaging sessions. GQDs are generally considered less toxic than other carbon-based nanomaterials due to their small size, hydrophilicity, and oxygen-containing surface functional groups, which enhance their aqueous dispersibility and reduce nonspecific membrane interactions.^68–70^ Nevertheless, the toxicity can vary significantly depending on the size, surface charge, dose, and surface functionalization. Ensuring a low-toxicity profile not only protects healthy cells and tissues but also allows for higher concentrations of GQDs to be administered when needed, thereby improving the imaging contrast without compromising safety. Therefore, optimizing GQD formulation to balance internalization efficiency with minimal toxicity is a fundamental step toward advancing their use in clinical diagnostics and image-guided therapies.^69^

On the other hand, low cellular uptake of GQDs in lung carcinoma cells can be overcome by functionalizing GQDs with targeting ligands or peptides that actively trigger endocytic pathways.^71^ Such moieties bind to overexpressed cell-surface receptors and induce receptor-mediated endocytosis, thereby increasing intracellular accumulation in the target cancer cells.^71^ For example, conjugating transferrin to GQDs exploits the high transferrin receptor (TfR) expression in A549 cells, leading to significantly higher uptake via clathrin-mediated endocytosis compared to untargeted GQDs.^72^ Likewise, attaching an RGD (Arg–Gly–Asp) peptide enables GQDs to bind integrins (e.g., α_vβ_3/5) commonly upregulated on lung cancer cells, facilitating integrin-mediated internalization and improved intracellular delivery of payloads.^73^ Other widely used targeting ligands include folic acid (to engage folate receptors) and hyaluronic acid (HA, targeting CD44 receptors), both of which markedly enhance GQD uptake in receptor-positive lung tumor cells via receptor-mediated endocytic routes.^74^ By leveraging these receptor-mediated uptake mechanisms, ligand-functionalized GQDs achieve higher endocytosis efficiency and greater selectivity for cancer cells (e.g., enhanced accumulation in tumor cells with reduced off-target uptake), ultimately improving imaging sensitivity and therapeutic efficacy.^71^

## 4 Conclusions

Tris­(2-aminoethyl)­amine functionalized graphene quantum dots (TREN N-GQDs) containing N atoms were synthesized by hydrothermal reaction of citric acid and TREN. Then, Ag, Pd, and Pt nanocomposites (MNPs/TREN N-GQDs) were prepared by using TREN N-GQDs as the reducing reagent and stabilizer. All synthesized materials were characterized by FT-IR, UV–vis, TEM, and EDX analysis. Interestingly, during the reduction of Ag, Pd, and Pt ions, especially the silver ion, was found to oxidize some OH groups in TREN N-GQDs to form ketones. This was detected by the formation of a new peak (1720 cm^–1^) above 1701–1720 cm^–1^ in FT-IR. The same was not observed for the other Pd and Pt ions. The performed femtosecond transient absorption spectroscopy measurements revealed that the excited-state lifetime was shortened in the presence of metal atoms as compared to TREN-GQDs. The z-scan results showed an enhanced nonlinear absorption that occurred by two-photon absorption due to the shortened lifetimes with the addition of metal nanoparticles.

Cell viability and *in vitro* imaging experiments demonstrated that the AgNPs/TREN N-GQDs nanocomposite showed potent cytotoxicity at low concentrations, effectively killing cancer cells. In contrast, PtNPs/TREN N-GQDs exhibits negligible impact on cell viability, indicating its high biocompatibility and suitability for bioimaging applications.

This study also used the DFT method to analyze TREN/N-GQDs and their nanocomposites containing Ag, Pd, and Pt nanoparticles. Structural optimizations in the gas phase revealed stable configurations, while simulations performed under aqueous conditions brought the theoretical data into better agreement with the experimental conditions. The obtained nanocomposites exhibited improved stability and optical properties due to increased reactivity, especially as indicated by the lower HOMO–LUMO energy gaps of AgNPs. These findings reveal the potential of metal nanoparticle-GQD composites in advanced materials science in areas such as photovoltaic applications and biosensor development. As a result of the combined evaluation of theoretical and experimental data, it was observed that not only the HOMO–LUMO energy gap but also the spatial distribution of molecular orbitals are decisive in the interactions of nanocomposites with biological systems. In particular, in the AgNPs/TREN N-GQDs nanocomposite, the high degree of delocalization of the HOMO and LUMO orbitals positioned close to the surface around the Ag atom allows this structure to interact more directly with cell membranes. This situation is consistent with the high cytotoxicity observed experimentally. On the other hand, although there is a similar energy gap in the PtNPs/TREN N-GQDs system, the fact that the molecular orbitals are largely localized on the GQD body and the Pt atom plays a limited role in these transitions reduces the interaction with cell structures and thus increases biocompatibility. Indeed, this system exhibited high intracellular accumulation and a low cytotoxicity profile in experimental imaging studies. This study also reveals that HOMO–LUMO energy gaps alone are not sufficient to explain the biological effect; the distribution of orbitals in space, charge density, and metal-center interactions are much more critical parameters in the interaction with biological systems.

In conclusion, the integration of Ag, Pd, and Pt nanoparticles into TREN N-GQDs opens new avenues for advancing nanocomposite technologies. While metal incorporation into the structure enhances the nonlinear absorption (NLA) responses, the inclusion of platinum (Pt) opens a pathway for bioimaging applications. The resulting enhancement in optical properties, combined with improved structural stability, makes these materials well-suited for a broad range of applications, from NLA-based technologies such as optical limiting to biological uses, including optical sensing and biomedical imaging. This versatility highlights their potential impact across diverse scientific and technological fields.

## Supplementary Material



## Data Availability

Data supporting this article have been included as part of the SI.

## Notes

Figure S1: EDX spectra of TREN N-GQDs; Figure S2: EDX spectra of AgNPs/TREN N-GQDs; Figure S3: EDX spectra of PdNPs/TREN N-GQDs; Figure S4: EDX spectra of PtNPs/TREN N-GQDs; Figure S5: photoluminescence emission of PtNPs/TREN N-GQDs; Figure S6: photoluminescence emission of AgNPs/TREN N-GQDs; Figure S7: photoluminescence emission of PdNPs/TREN N-GQDs; Figure S8: transient absorption spectra of TREN N-GQDs; Figure S9: transient absorption spectra of Ag-TREN N-GQDs at 400 nm pump wavelength and 120 fs pulse duration; Figure S10: transient absorption spectra of Pd-TREN N-GQDs; and Figure S11: transient absorption spectra of Pt-TREN N-GQDs (PDF)
