# Supplementary material for: Plasmonic Enhanced Nonlinear Absorption of Tris(2-aminoethyl)amine (TREN) Functional Ag, Pt, and Pd Nanoparticle-GQDs Complexes and Their Evaluation as Potential Bioimaging Applications
Source: ACS Appl Bio Mater. 2025 Jul 1;8(7):6261–77. doi: 10.1021/acsabm.5c00750 (PMC12284890; doi:10.1021/acsabm.5c00750)
Supplement: Supplementary file 1 [file mt5c00750_si_001.pdf]

Supporting Information

for

Plasmonic Enhanced Nonlinear Absorption of Tris(2-aminoethyl)amine (TREN) functional Ag, Pt and Pd Nanoparticle-GQDs Complexes and Their Evaluation as Potential Bioimaging Applications

Bekir Asilcan Unlu[a,b], Esen Kirit[c], Dogantan Celik[c,d], Elif Akhuseyin Yildiz[a,e], Ahmet Karatay[a,*], Bahadir Boyacioglu[f], Hüseyin Ünver[g], Açelya Yilmazer[c,d,*], Mustafa Yıldız[h,*], Ayhan Elmali[a,*]

[a] *Department of Engineering Physics, Faculty of Engineering, Ankara University, 06100, Beşevler, Ankara, Türkiye*
[b] *Graduate School of Natural and Applied Sciences, Ankara University, Ankara, Türkiye.*
[c] *Department of Biomedical Engineering, Faculty of Engineering, Ankara University, 06830, Ankara, Türkiye*
[d] *Stem Cell Institute, Ankara University, 06520, Ankara, Türkiye*
[e] *Institute of Artificial Intelligence, Ankara University, TR-06100, Ankara, Türkiye*
[f] *Vocational School of Health Services, Ankara University, Kecioren, 06290, Ankara, Türkiye*
[g] *Department of Physics, Faculty of Science, Ankara University, 06100Besevler, Ankara, Türkiye*
[h] *Department of Chemistry, Faculty of Arts and Sciences, Çanakkale Onsekiz Mart University, TR-17100 Çanakkale, Türkiye.*

Corresponding authors: akaratay@eng.ankara.edu.tr (A. KARATAY), ayilmazer@ankara.edu.tr (A. YILMAZER), myildiz@comu.edu.tr (M. YILDIZ), elmali@eng.ankara.edu.tr (A. ELMALI)

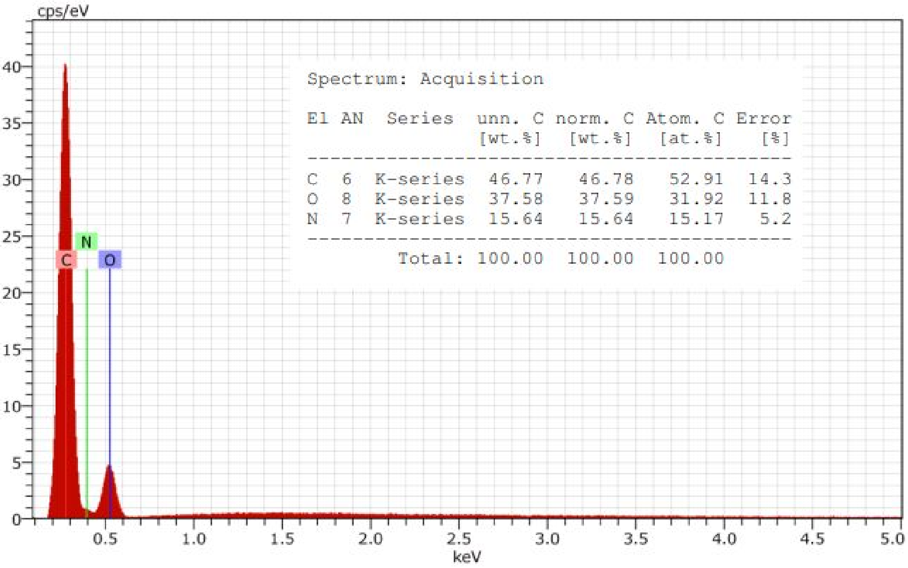

Figure S1. EDX spectra of TREN N-GQDs

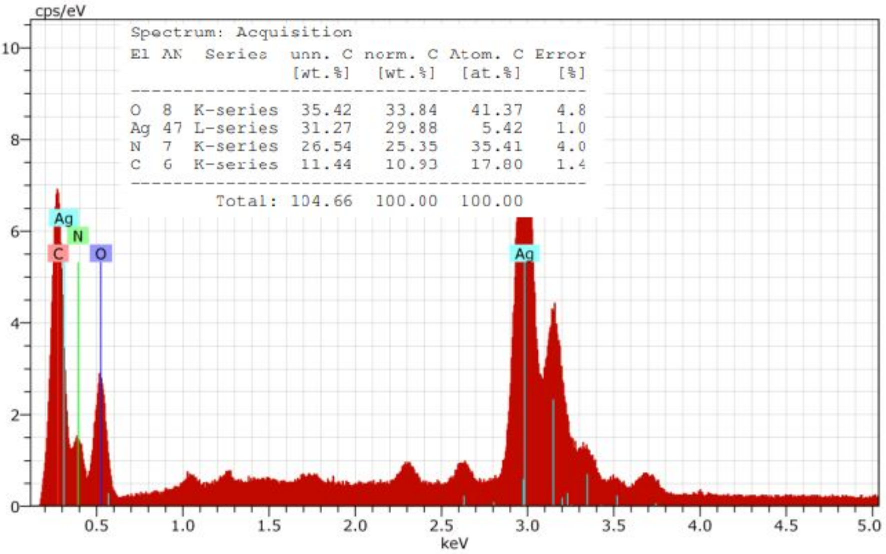

Figure S2. EDX spectra of AgNPs/TREN N-GQDs

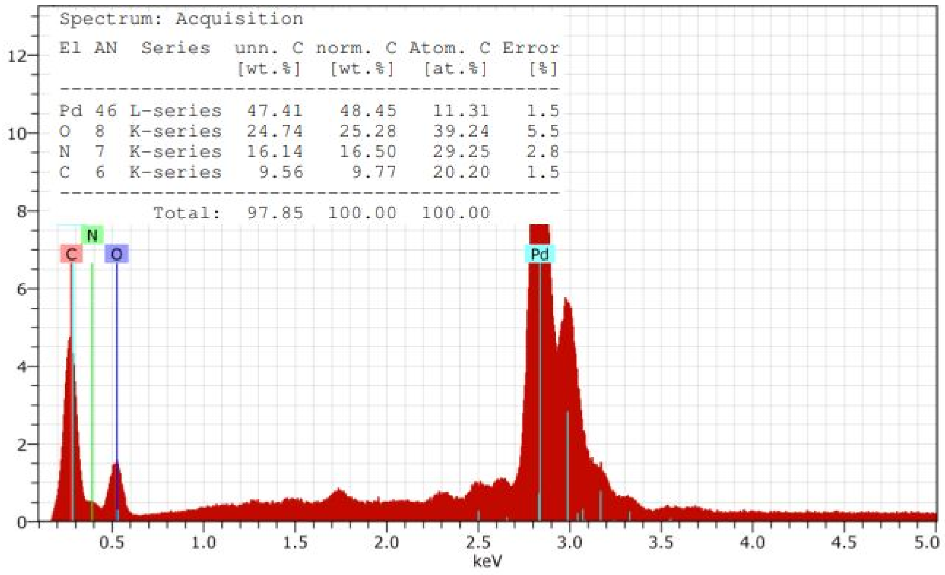

Figure S3. EDX spectra of PdNPs/TREN N-GQDs

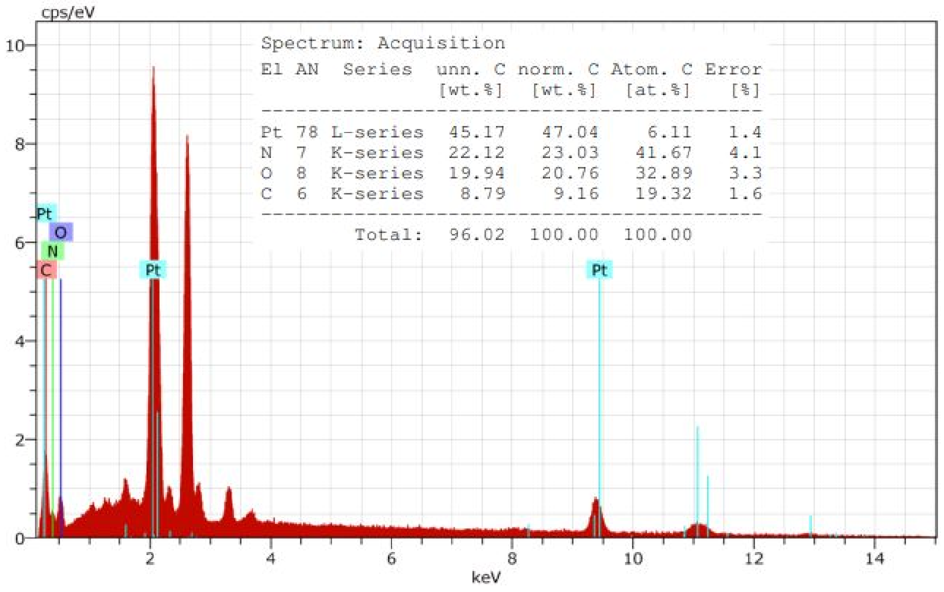

Figure S4. EDX spectra of PtNPs/TREN N-GQDs

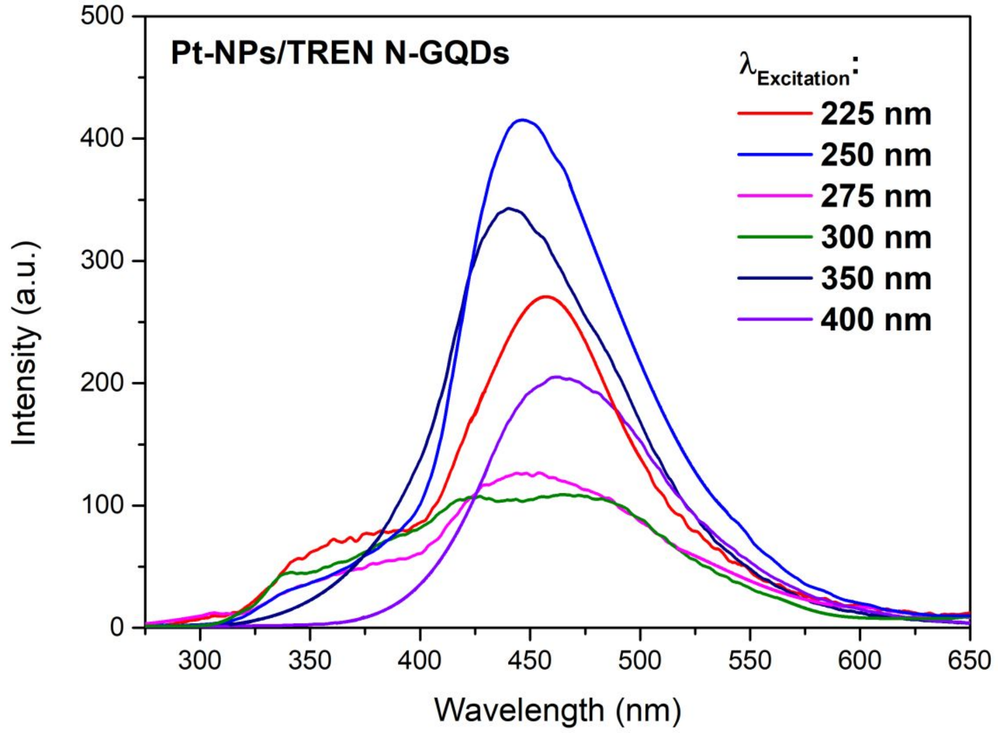

Figure S5. Photoluminescence emission of PtNPs/TREN N-GQDs at different excitation wavelengths

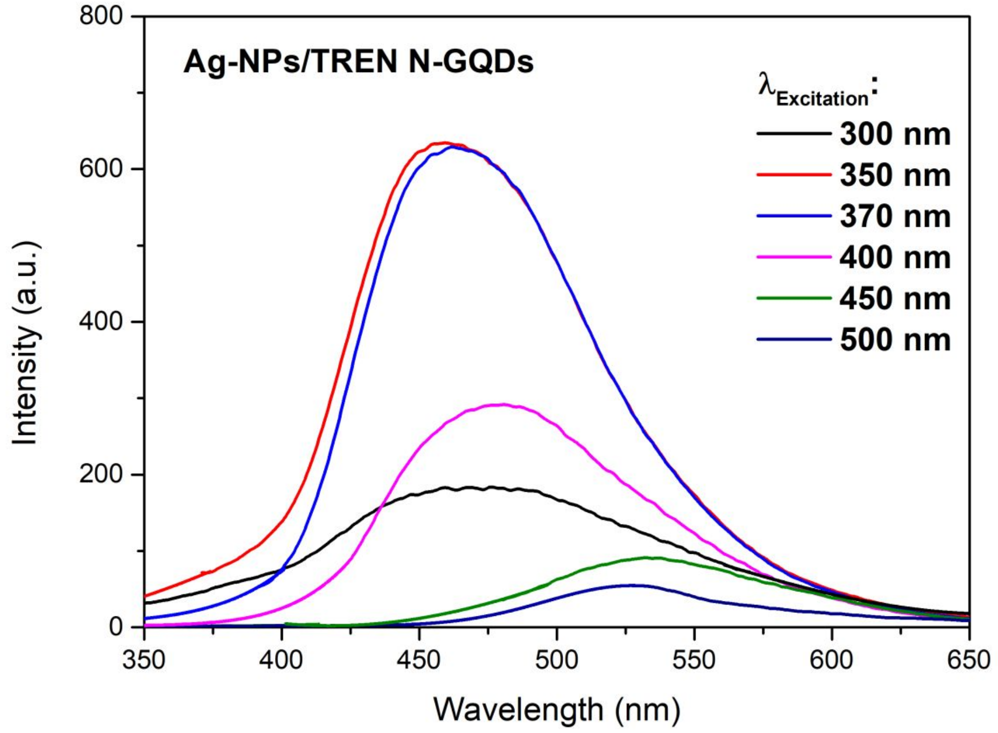

Figure S6. Photoluminescence emission of AgNPs/TREN N-GQDs at different excitation wavelengths

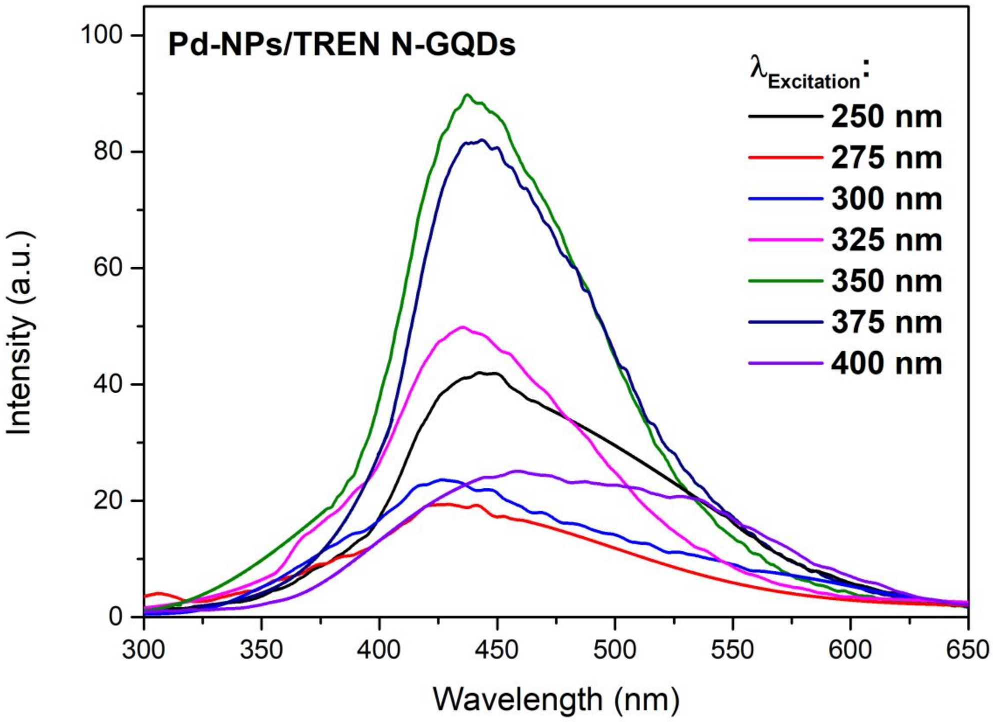

Figure S7. Photoluminescence emission of PdNPs/TREN N-GQDs at different excitation wavelengths

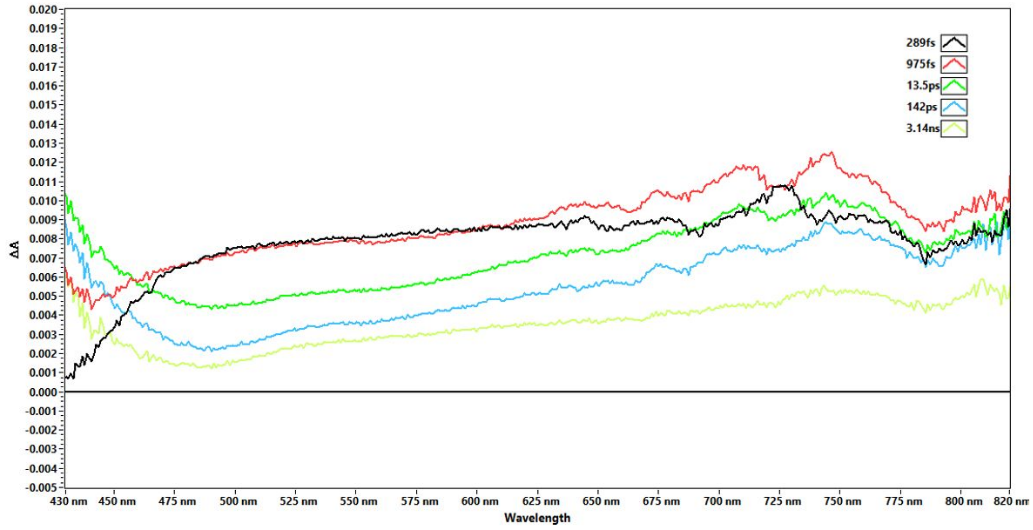

Figure S8. Transient absorption spectra of TREN N-GQDs at 340 nm pump wavelength and 120 fs pulse duration

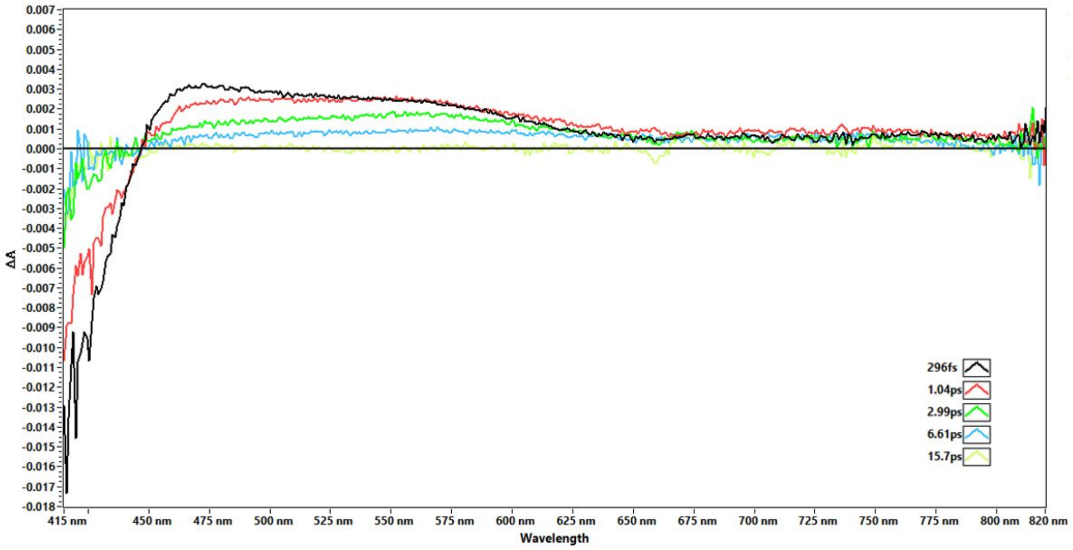

Figure S9. Transient absorption spectra of Ag-TREN N-GQDs at 400 nm pump wavelength and 120 fs pulse duration

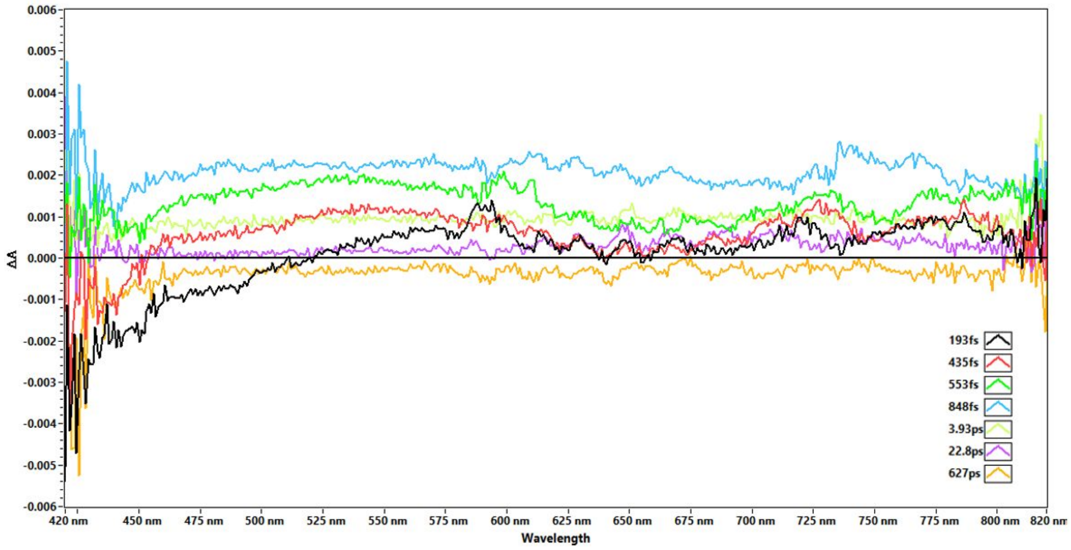

Figure S10. Transient absorption spectra of Pd-TREN N-GQDs at 300 nm pump wavelength and 120 fs pulse duration

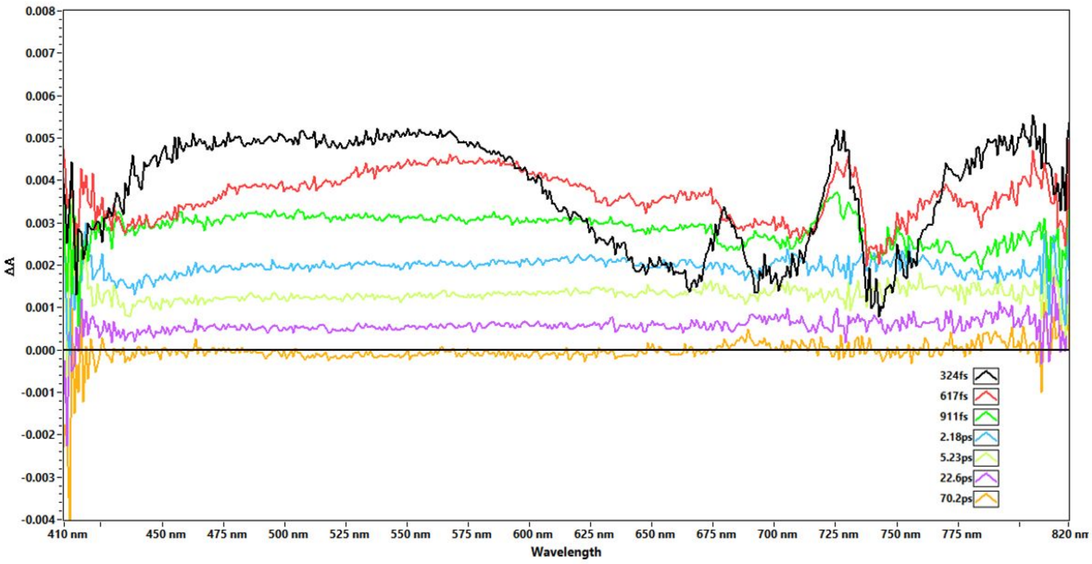

Figure S11. Transient absorption spectra of Pt-TREN N-GQDs at 340 nm pump wavelength and 120 fs pulse duration
